# Correlation between reading time and characteristics of eye fixations and progressive lens design

**DOI:** 10.1371/journal.pone.0281861

**Published:** 2023-03-27

**Authors:** Pablo Concepcion-Grande, Eva Chamorro, Jose Miguel Cleva, Jose Alonso, Jose Antonio Gómez-Pedrero

**Affiliations:** 1 Clinical Research Department, Indizen Optical Technologies, Madrid, Spain; 2 Applied Optics Complutense Group, Optics Department, Optics and Optometry Faculty, Complutense University of Madrid, Madrid, Spain; The Ohio State University, UNITED STATES

## Abstract

**Objective:**

The purpose of this study is to evaluate reading time and characteristics of fixations at different distances when looking through different areas of progressive power lenses (PPL) with different power distributions by means of eye-tracking technology.

**Method:**

A wearable eye tracker system (Tobii-Pro Glasses 3) was used to record the pupil position of 28 PPL subjects when reading at near and distance vision while using 3 different PPL designs: a PPL optimized for distance vision (PPL-Distance), a PPL optimized for near vision (PPL-Near) and one of them balanced for a general use (PPL-Balance). Subjects were asked to read out loud a text displayed on a digital screen located at 5.25m and 0.37m when they were looking through the central and peripheral regions of each PPL. Reading time, total duration of fixations, and the number of fixations were analyzed for each reading condition and PPL. Statistical analysis was carried out using Statgraphics Centurion XVII.II Software.

**Results:**

The analysis of eye movements at distance-reading vision showed a statistically significant lower reading time (p = 0.004) and lower total duration of fixations (p = 0.01) for PPL-Distance. At near-reading vision, PPL-Near provided statistically significant lower reading time (p<0.001), lower total duration of fixations (p = 0.02), and less fixation count(p<0.001) in comparison with PPL-Balance and PPL-Distance.

**Conclusions:**

Reading time and fixations characteristics are affected by the power distribution of a PPL. A PPL design with a wider distance region provides better distance-reading performance while a PPL with a wider near area performs better at a near-reading task. The power distribution of PPLs influences the user performance at vision-based tasks. Thus, to provide the user with the best visual experience, PPL selection must consider user needs.

## Introduction

Reading is a highly complex task that involves the integration of oculomotor, sensory, cognitive, and attentional functions [1]. A normal reading is comprised of accurate, rhythmical and reflexively executed sequences of saccade eye movements separated by brief pauses called fixations. Saccades are fast movements of different amplitudes and fixations are periods in which the eyes remain relatively still between saccades [2].

Video-based eye tracking (ET) allows to record and analyze eye movements [3]. Most ET devices use infrared light to illuminate the user’s eyes, record the position of the pupil with a camera and calculate eye movements from the image data [4]. Thanks to these systems, it has been possible to study how eye movements during reading are affected by different factors such as reader’s proficiency, text characteristics [5], accommodative response, vergences or blur [6, 7], In this sense, studies evaluating reading in subjects with reading difficulties or different reading skills levels [8–15] have shown that skilled readers had fewer and shorter fixations when reading than non-skilled readers. Other studies have demonstrated that blur such as non-compensated refractive error is associated with variations on reading ability. Garzia et al [16], simulated 2.00D of bilateral hyperopia on 19 adult subjects and reported an increase of 11% in reading time. Young and Hopkins et al [17, 18], associated myopia with improvements in reading abilities. Wills et al [19] reported a reduction of reading speed by up to 24% when simulating astigmatism blur as low as 1.00D at 90°. A similar outcome was reported by Casagrande et al [20], who showed a reduction in reading speed by 35% with a simulated astigmatic blur of 0.75D at 90°.

Comparably to blur produced by non-compensated refractive errors, blur induced at the lateral regions of PPLs can affect reading performance. A PPL is a multifocal lens characterized by having a smooth increase in spherical power from the upper part of the lens to the bottom. This power variation through the vertical axis allows presbyopic spectacle wearers to see clearly at all distances by changing the gaze position [21]. This power variation along the vertical axis of the lens results in the appearance of unwanted astigmatism in the lateral regions of the lens [22]. Unwanted astigmatism limits the area of the undistorted field of view of users, and its gradient is also responsible for swimming effects, unwanted distortion, and dizziness in dynamic visual conditions [23].

Several studies have used ET to analyze how eye movements during reading are affected by PPLs. The study of Han et al [24, 25] used ET techniques to determine eye movements differences between PPLs and single-vision lenses. Concepcion et al [26] analyzed the differences in visual behavior of different visual areas of the PPLs during reading on a computer screen. Rifai et al [27] evaluated eye and head movements during driving in PPL users in comparison with non-PPL users. All of them showed that blur on the lateral regions of PPLs affects reading. However, the use of eye movements to differentiate among PPLs with different power distributions has not been widely spread. For this reason, in the present study, we propose the use of an ET system as a potential tool to evaluate differences between PPLs. The main objective of this study is to evaluate and compare reading time and characteristics of fixations when using three PPLs with different power distributions when performing a distance-reading and near-reading tasks.

## Methods

### Study design

A prospective observational longitudinal double-masked study was carried out to evaluate reading time and characteristics of fixations when reading with 3 different types of PPL. The study followed the tenets of the Declaration of Helsinki. Full approval for the study was obtained from the Hospital Clínico San Carlos Ethics Committee’s (CEIC) Review Board (15/361-P). All subjects provided their written informed consent before starting the study and, at the end of the trial, subjects were compensated with two pair of eyeglasses.

### Participants

The study sample was comprised of presbyopic subjects of both genders aged over 45 and with at least 6 months prior experience of PPL wear. The inclusion criteria were: 1) Refractive error range between -6.00D and +4.00D with astigmatism lower or equal to 2.50D. 2) Near addition between +1.00D and +3.00D. 3) Best-corrected visual acuity (VA) better than 0.10logMAR monocular and 0.05logMAR binocular. 4) Anisometropia lower than 1.50D. Subjects were excluded if they had medical pathologies which could affect vision, significant binocular vision anomalies, ocular pathologies, or if they had been in any pharmacological treatment that could affect the subjective assessment or the visual function. The sample size was estimated based on data from a pilot study carried out with 5 participants with the same inclusion criteria mentioned above. The calculation was done using GRANMO sample size calculator version 7.12 (Institut Municipal d’Investigació Mèdica, Spain). The obtained estimated sample size was 27 subjects, assuming an alpha risk of 0.05, a beta risk of 0.1 in two-tailed testing, and a dropout rate of 15%.

### Procedure

All subjects underwent a comprehensive optometric evaluation to check whether they meet the inclusion criteria. The visual exam consisted of VA testing using PVVAT test (Precision Vision, La Salle, III), subjective refraction at distance and near vision, stereoacuity evaluation using Titmus test, cover test, Worth test, and ocular motility analysis. Once optometrists confirmed the participant met the inclusion criteria, measurement of fitting parameters and position of wear (pupillary distance, segment height, pantoscopic tilt, back vertex distance, and frame wrap angle) for the ET eyeglasses with the clip-on attached were measured, and PPL lenses were ordered. Measurements of eye movements for the 3 different types of PPL at different working distances were obtained in an independent visit with a duration of 2 hours. Two-minute breaks between each measurement were done to minimize participants’ fatigue.

### Progressive power lenses

**Fig 1:**
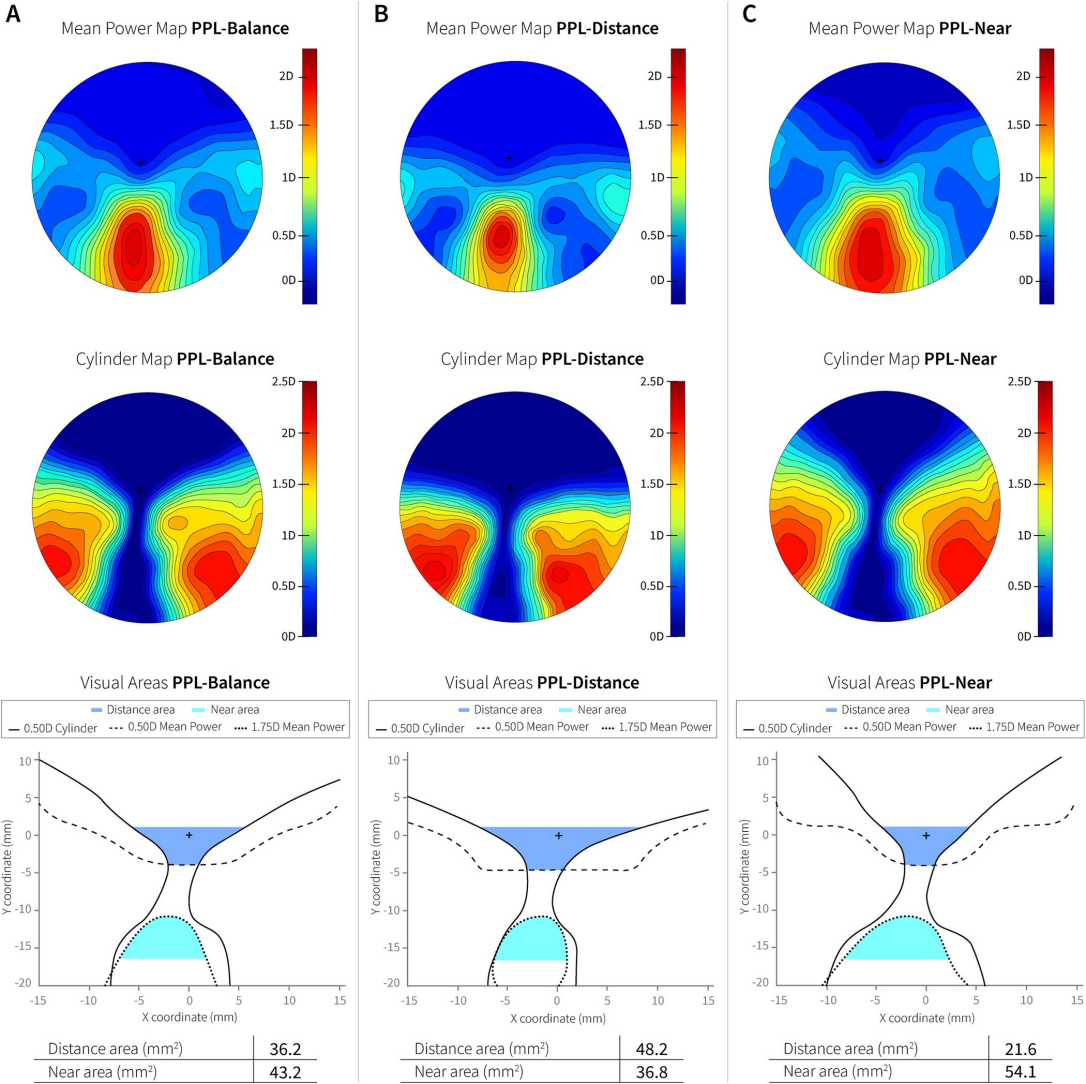
Mean power, cylinder power maps distribution, and visual areas according to Sheedy’s criteria for plano addition 2.00D prescription using standard parameters of the PPLs tested in the research. A) PPL-Balance. B) PPL-Distance. C) PPL-Near.

**Fig 2:**
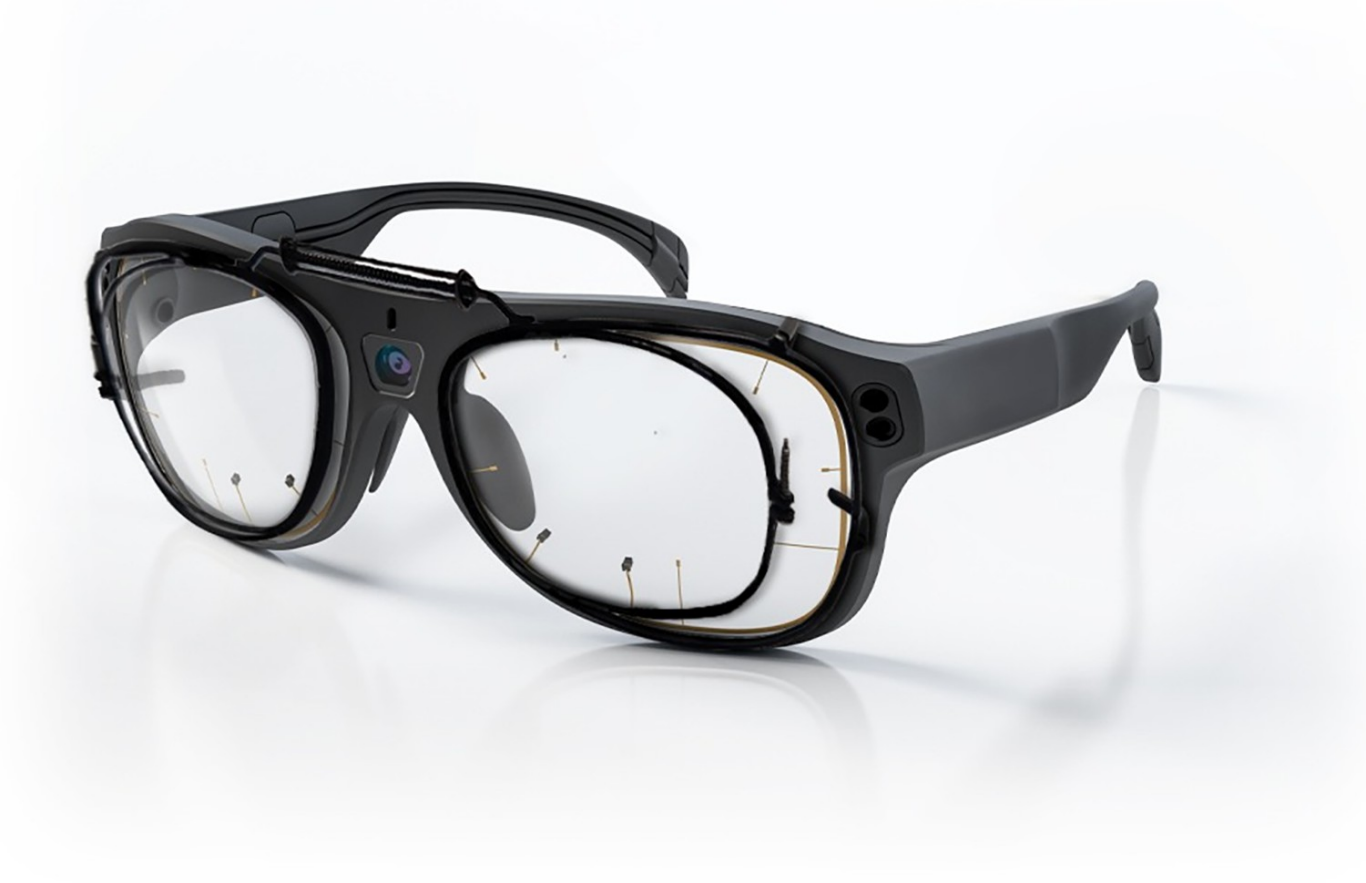
Picture of the metallic frame developed ad-hoc for this study attached to Tobii Glasses PRO 3.

Three free-form PPL designs individualized with different power distribution maps were developed ad-hoc for this research: 1) PPL-Balance, the control lens (Endless Steady Balance, IOT, Madrid, Spain), 2) PPL-Distance, a lens with a wider visual area for distance vision (Endless Steady Distance, IOT, Madrid, Spain) and 3) PPL-Near, a lens with wider visual area for near vision (Endless Steady Near, IOT, Madrid, Spain). Mean power, cylinder power maps distribution, Sheedy contours and visual areas according to Sheedy’s criteria [28] for a plano addition 2.00D prescription using standard parameters are shown in Fig 1. Lenses were mounted in a specific clip-on (developed ad-hoc) that was attached to the front of the ET eyeglasses. This set up allowed the direct recording of the pupils without any interference from the progressive lenses (Fig 2).

Lenses were calculated using fitting parameters and position of wear of the ET with the clip-on using advanced optical design software (Freeform designer, IOT, Madrid, Spain) to reduce oblique aberrations and maintain fields of view stable independent of each subject’s prescription and/or addition power for all gaze directions. According to Sheddy’s criteria [28], the PPL-Distance lens has a 29% wider field of view and the PPL-Near lens a 22% narrower field of view in the distance zone compared to the control lens (PPL-Balance). In the near zone, compared to the control lens, the PPL-Near has a 27% wider field of view and the PPL-Distance a 12% narrower field of view.

### ET evaluation

A wearable ET system with a sample rate of 50 Hz (Tobii-Pro Glasses 3, Tobii AB, Sweden) was used to record binocular pupil position. Recordings were done while subjects were reading at distance and near distances. Pupil position data were processed to obtain fixations and their characteristics using Tobii Pro Lab Software (Tobii AB, Sweden) and Tobii I-VT fixation filter [29, 30] setting a velocity threshold of 10°/s to classify fixations.

**Fig 3:**
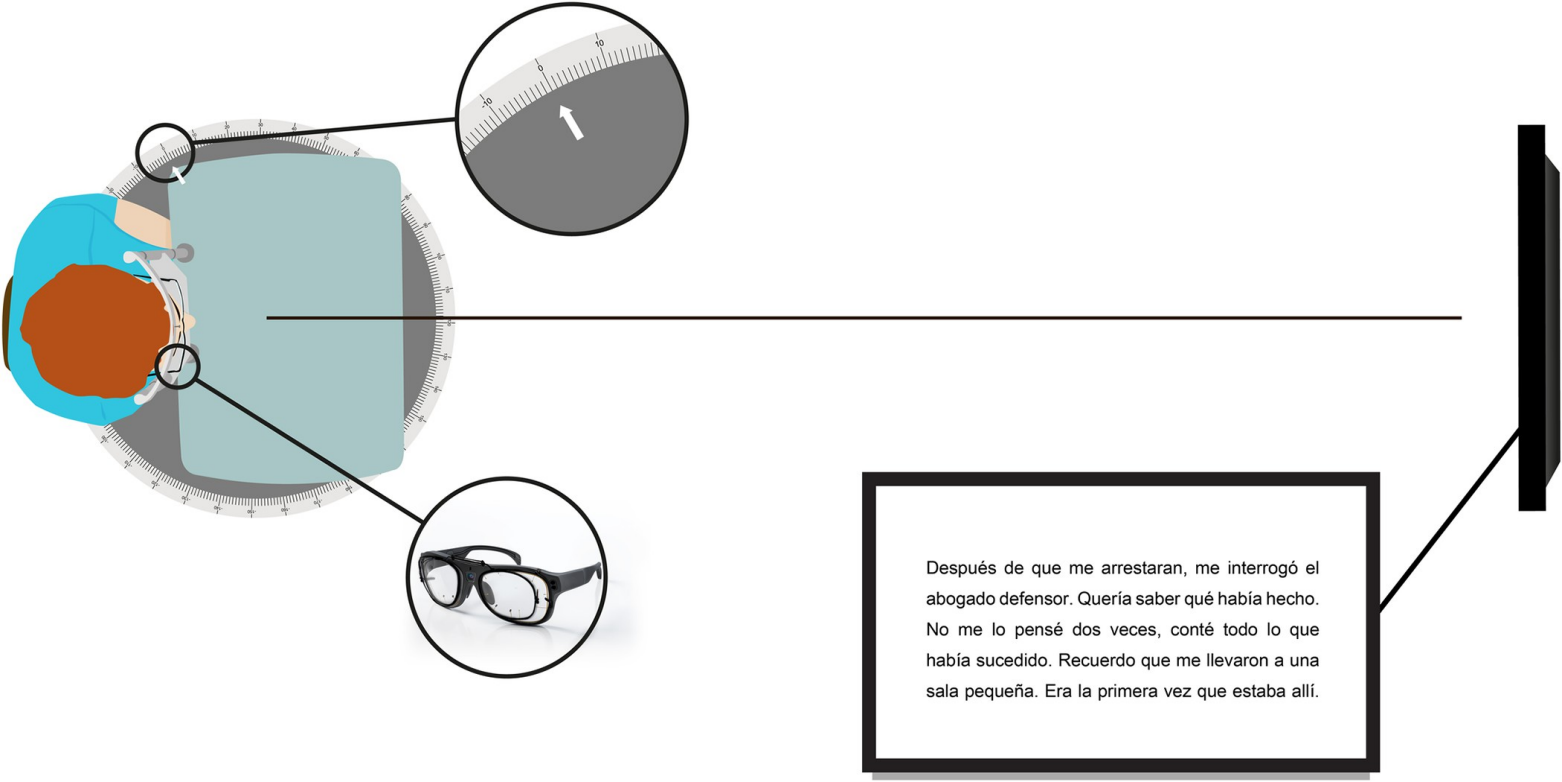
Scheme of the evaluation of reading performance using ET technology at distance reading vision.

Distance-reading performance was recorded while reading a text shown on a screen monitor (Aus LCD Monitor VP228HE 21.5”) located at 5.25m. The text subtended 4.2° horizontally and 2.3° vertically and was composed of 5 text lines having a font size of 0.4logMAR. To evaluate a greater area, subjects were rotated into 3 different orientations: on-axis and two off-axis reading positions (10° and 15°). Off-axis rotations were induced using a rotation platform with a chinrest to prevent head mobility (Fig 3). For each position, subjects were asked to read out loud the text displayed on the screen with the 3 PPLs. Therefore, nine reading texts for the different experimental conditions (3 PPLs and 3 rotations) were used in a randomized order.

**Fig 4:**
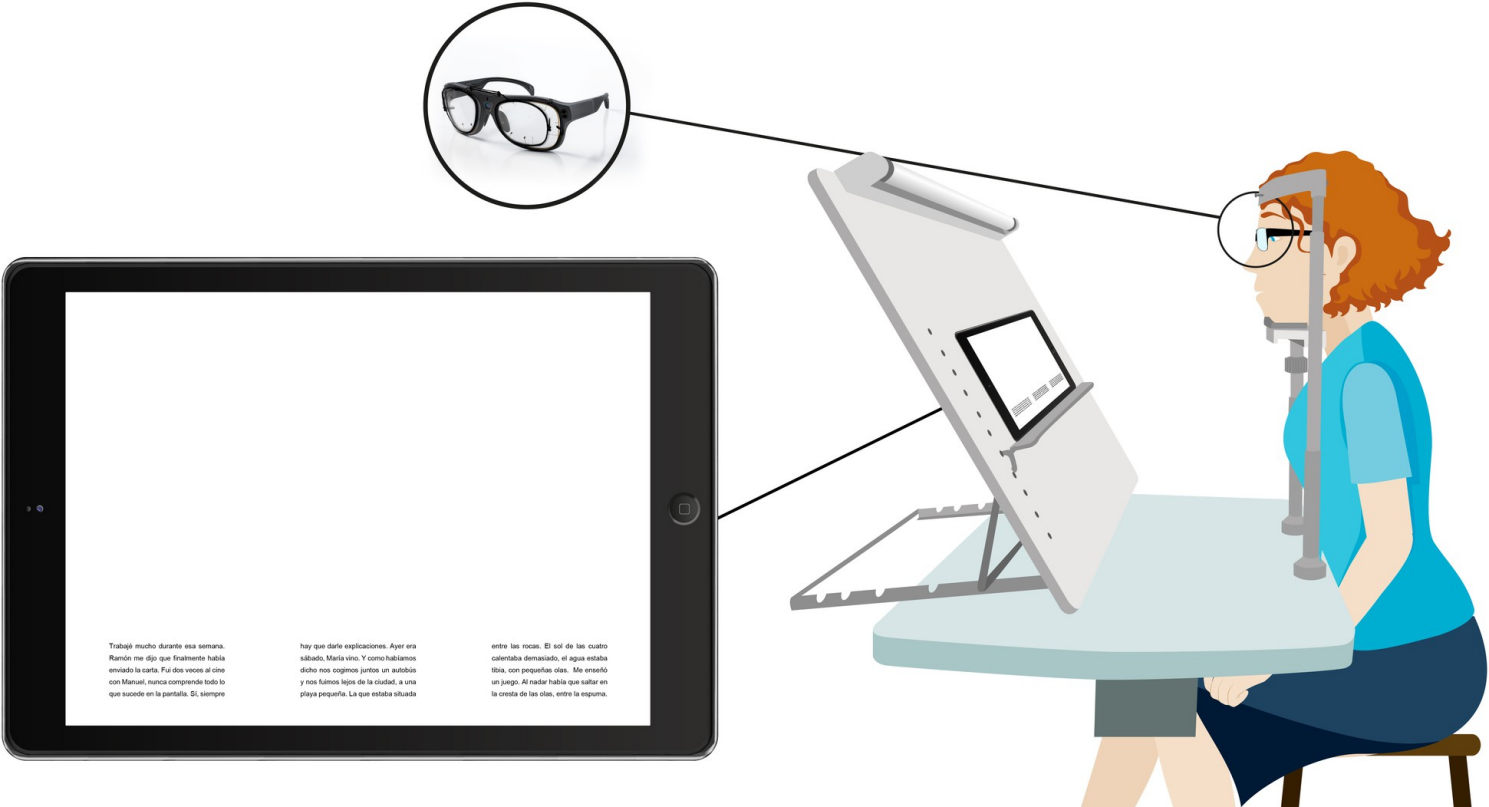
Scheme of the evaluation of reading performance using ET technology at near-reading vision.

Near-reading performance was assessed at 0.37m using 3 different reading texts (randomized for each PPL) displayed on a screen (Microsoft Surface PRO 4 12.3"). Each reading text was composed of three columns with five text lines each with a font size of 0.4logMAR and a viewing angle of 28° horizontally and 3.6° vertically. A table with a chinrest was used to prevent head movements and ensure subjects used the PPL central and lateral regions (Fig 4).

The homogeneity of the texts used for distance and near reading was analyzed by a linguistic analysis ensuring that each text for each reading condition presented similar number of words, syllables, single-syllable words, average characters per word and ISFZ index [31, 32] (Table 1). Additionally, a pilot study with 10 emmetropic participants without wearing lenses was carried out to ensure that there were no significant differences between each text for each reading condition in reading time, total duration of fixations, and fixation count.

**Table 1:** Characteristics of texts (words count, syllable count, single syllable word count, average characteristics per word and ISFZ index) used for reading assessment at distance and near vision.

	Words count	Syllable count	Single-syllable word count	Average characters per word	ISFZ index
**Distance-reading vision**	43 ± 2	82 ± 3	19 ± 1	4,4 ± 0,2	84 ± 7
**Near-reading vision**	32 ± 2	60 ± 3	13 ± 2	4,4 ± 0,3	85 ± 6

### Statistical analysis

Statgraphics Centurion XVI.II Software was used in all the statistical analyses carried out in this research. Differences in eye movements depending on the power distribution maps of PPLs and orientations for distance and columns for near-reading vision were analyzed using a multifactorial ANOVA test, the statistical power was set at 0.8 and the level of significance at 0.05. To find differences in which means are significantly different from each other, a LSD post-hoc test was performed. Analyzed variables were reading time, fixation time, and fixation count.

## Results

### Sample characteristics

The sample was composed of 28 subjects (17 men and 11 women) of mean age 55±7 (from 46 to 64 years). According to the optometric evaluation the average mean refractive error was -0.64±2.20 D (From -5.38 to +2.25). There were 14 subjects with myopia, 10 hyperopic subjects and 4 of them were emmetropic subjects. The addition power of the participants ranged from 1D to 2.50D with and average value of 1.96±0.47 D.

### Distance-reading vision

**Fig 5:**
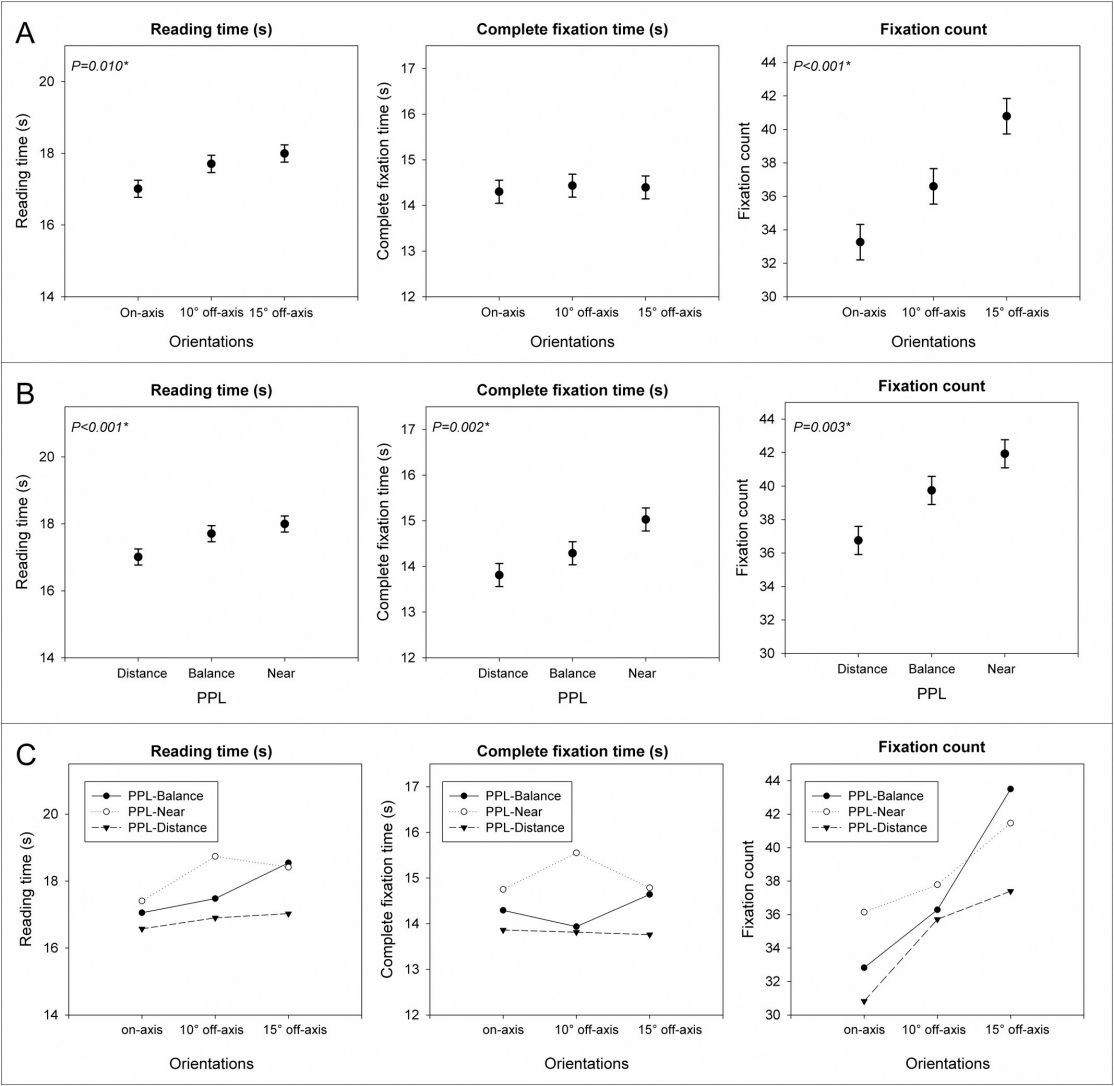
Variations in reading time, fixation time and fixation count depending on the PPL (A), the orientation (B) and the interactions of PPL and orientation (C) for distance-reading task.

Statistically significant differences were found between PPLs for distance-reading vision. When subjects were using PPL optimized for distance vision, reading time was reduced and the total duration of fixations and number of fixations was decreased in comparison with PPL-Balance and PPL-Near. Statistically significant higher reading time and number of fixations were found for off-axis orientations in comparison with on-axis (Fig 5 and Table 2).

**Table 2:** Detailed statistics for Fig 5.

	**ANOVA test for lens design**				**LSD Comparisons for lens design**		
	**Df**	**Mean square**	**F-ratio**	**P-value**	**Balance/Near**	**Balance/Distance**	**Distance/Near**
					**(Diff limit)**	**(Diff+/-limit)**	**(Diff+/-limit)**
**Reading time**	2	39.31	8.89	<0.001*	-0.49*+/-*0.64	0.86*+/-*0.64*	-1.35*+/-*0.64*
**Fixation time**	2	31.62	6.45	0.002*	-0.74*+/-*0.68*	0.48*+/-*0.68	-1.22*+/-*0.68*
**Fixation count**	2	333.68	6.16	0.003*	-0.93*+/-*2.25	2.89*+/-*2.25*	-3.8*+/-*2.25*
	**ANOVA test for orientation**				**LSD comparisons for orientation**		
	**Df**	**Mean square**	**F-ratio**	**P-value**	**On-axis/10° off-axis**	**On-axis/15° off-axis**	**10°/15° off-axis**
					**(Diff+/-limit)**	**(Diff+/-limit)**	**(Diff+/-limit)**
**Reading time**	2	21.47	4.86	0.01*	-0.69*+/-*0.64*	-0.98*+/-*0.64*	-0.29*+/-*0.64
**Fixation time**	2	0.39	0.08	0.924	-0.13*+/-*0.68	-0.09*+/-*0.68	0.04*+/-*0.68
**Fixation count**	2	1193.90	22.03	<0.001*	-3.34*+/-*2.25*	-7.52*+/-*2.25*	-4.19*+/-*2.25*

Multifactorial ANOVA test with pos-hoc comparisons using LSD

* Shows significance at the 0.05 level.

### Near-reading vision

**Fig 6:**
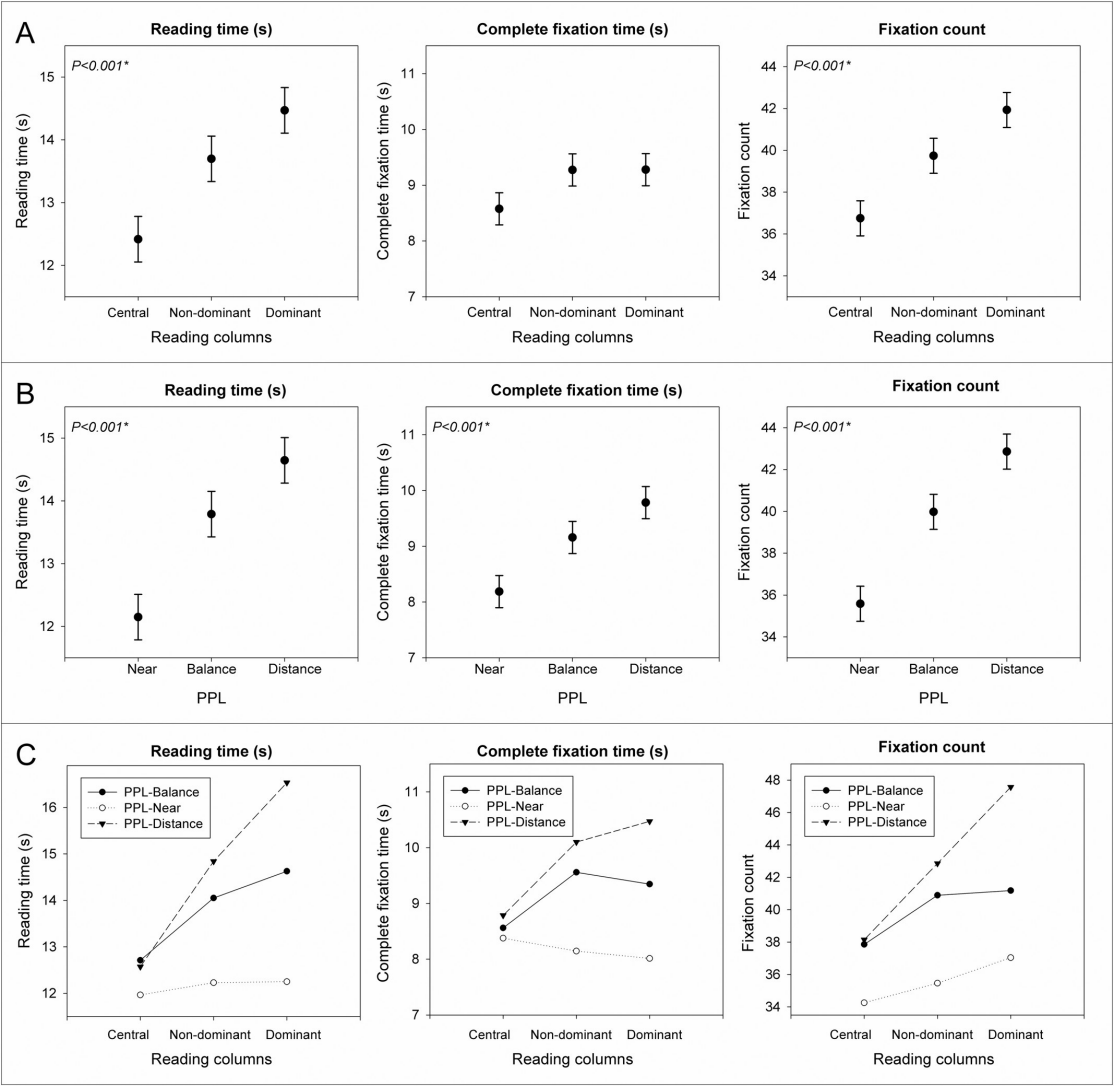
Variations in reading time, fixation time and fixation count depending on the PPL (A), the orientation (B) and the interactions of PPL and orientation (C) for near-reading task.

Statistically significant differences were found between PPLs for near-reading vision. When subjects were using PPL optimized for near vision, reading time was decreased, the total duration of fixations was decreased, and the number of fixations was reduced in comparison with PPL-Balance and PPL-Distance. Statistically significant lower reading time, lower fixation time and less fixation count were found for central column in comparison with peripheral columns (Fig 6 and Table 3).

**Table 3:** Detailed statistics for Fig 6.

	**ANOVA test for lens design**				**LSD Comparisons for lens design**		
	**Df**	**Mean squeare**	**F-ratio**	**P-value**	**Balance/Near**	**Balance/Distance**	**Distance/Near**
					**(Diff+/-limit)**	**(Diff+/-limit)**	**(Diff+/-limit)**
**Reading time**	2	135.30	12.24	<0.001*	1.64+/-0.81*	-0.86+/-0.81*	2.50+/-0.81*
**Fixation time**	2	54.27	7.79	<0.001*	0.97+/-0.58*	-0.63+/-0.58*	1.60+/-0.58*
**Fixation count**	2	1127.08	19.12	<0.001*	4.40+/-2.36*	-2.88+/-2.36*	7.27+/-2.36*
	**ANOVA test for reading columns**				**LSD Comparisons for reading columns**		
	**Df**	**Mean squeare**	**F-ratio**	**P-value**	**Center/Dominant (Diff+/-limit)**	**Center/ Non-dominant (Diff+/-limit)**	**Dominant/Non- dominant (Diff+/-limit)**
**Reading time**	2	90.37	8.18	<0.001*	-2.05+/-0.81*	-1.28+/-0.81*	0.77+/-0.81
**Fixation time**	2	13.67	1.96	0.143	-0.70+/-0.58*	-0.70+/-0.58*	0.01+/-0.80
**Fixation count**	2	567.62	9.63	<0.001*	-5.18+/-2.36*	-2.99+/-2.36*	2.19+/-2.36

Multifactorial ANOVA test with pos-hoc comparisons using LSD

* Shows significance at the 0.05 level.

## Discussion

The results of this study show that when participants are induced to look through different areas of the lens, there are variations in reading time, total duration of fixations, and fixation count depending on the visual area of the PPL. A PPL design with a wider distance visual area provides lower reading time and less fixation count than PPL-Balance and PPL-Near, improving reading at distance vision. Similarly for near-vision reading, a PPL design with a wider near area provides lower reading time, lower total fixation time, and less fixation count than PPL-Balance and PPL-Distance, improving reading at near vision. Also, it has been observed the influence of peripheral refractive errors of the PPLs in reading performance. More specifically, reading time and fixation count was higher at off-axis positions in comparison with on-axis positions both at near and distance reading tasks.

These results are in accordance with other studies that have used ET systems to evaluate the influence of PPLs in eye and head movements. In the study from Han et al [25], the differences between one single-vision lens and two PPLs on 11 presbyopic subjects was studied. Subjects had to read out loud a copy text printed in a standardized single page located along the subject’s midline at 0.60m. Eye and head positions were analyzed with the ISCAN integrated eye-head movement computer base system. The results showed that reading time and number of fixations were higher when subjects were using PPLs in comparison with single-vision lenses. Another study from Concepcion et al [26], recorded eye movements using a Tobii X3-120 ET system on 38 presbyopic subjects while using two different PPLs. Subjects were asked to read out loud a text on a computer screen located at 0.67m, at on-axis and off-axis viewing position. Results showed that complete fixation time and fixation count increases in the lateral areas of the lens where unwanted astigmatism increases. Both studies indicated that unwanted astigmatism in the lateral areas of the lens affect eye movements during reading. In accordance with them, our study shows that when visual fields are constrained, the reading time, complete fixation time and fixation count increase. In addition, our results show that when using the central area of the lens, with less refractive errors than the periphery, reading time, complete fixation time and fixation count decrease.

According to previous studies, it is clearly stablished that astigmatism affects reading. Wolffsohn et al [33] evaluated reading speed with different astigmatic blurs on 21 presbyope subjects from 50 to 69 years old using reading texts. Results showed that reading speed decreases with unwanted astigmatism mainly for astigmatic powers greater than 3D. Wills et al [19] used an ET system (Visiograph III) to calculate reading speed on 30 young subjects (18 to 33 years old) while reading a text at 40cm with simulated astigmatic blurs. Results showed a reduction on reading speed by up to 24% for astigmatic powers lower than 2D. Casagrande et al [20] calculated the reading time using a microphone to record the voice of 23 adults (20 to 32 years old). Results showed that reading speed was reduced by up to 35% with astigmatic blur of -0.75D at 90 and -1.50D at 90° and 180°. These results are consistent with our findings on reading being affected by the field of view of the PPLs.

There are some studies where reading pattern has evaluated between good and poor readers. Bucci et al [10] studied how fixations are affected during reading on 12 dyslexic and 19 non dyslexic children using an ET with a sample rate of 300Hz. Subjects were asked to read aloud a text composed by 4 lines located at 58 cm. Results showed that dyslexic children had more and longer fixations than non-dyslexic children. Griffin et al [15] compared fixation count and reading time of 13 poor readers and 13 good readers of an age between 9 and 11 years old using a video camera. The analysis showed that poor readers needed more fixations per word and more time than those participants with normal reading skills. A similar result was obtained by Landerl et al [34] using an Eye Link 1000 ET on 137 third and fourth graders classified as good or poor readers. Results showed that poor readers had higher number of fixations and total fixation time than those participants classified as good readers. Poor readers need more and longer fixations than good readers, resulting in longer reading times. Our study shows differences between PPLs in the same variables than these studies, but in a different magnitude. Those PPL with a narrower field of view for the specific reading-task produce more and longer fixations, and thus higher reading time. This result suggests that there is a deterioration of reading due the power distribution of a PPL.

For future studies, it would be interesting to incorporate some modifications in the set-up of the experiment that could provide additional information. First, regarding the evaluation of distance-reading performance, it would be interesting to evaluate wider area in a more natural position. In the present study, we used a screen of 21.5” located at 5.25m from the subject’s eye, which means that the horizontal and vertical visual field subtends 4.2° and 2.3° respectively in each distance-reading condition. To evaluate a greater distance area, it was necessary to rotate subjects in 3 different locations increasing the complexity of the experiment. To simulate a more natural conditions, it would be necessary to use a bigger screen with a wider visual field without the need of using a rotation platform. Similarly, it would be interesting to evaluate reading performance and ocular movements without the restriction of a chinrest. Finally, the previous PPL design used by each participant was not registered in our study due to the variability of options in the market. In future studies, it could be interesting to include this analysis to study the influence of adaptation to prior design in ocular movements using different PPL designs.

To conclude, reading time and characteristics of fixations change between PPLs with different power distributions for distance-reading and near-reading tasks. Thus, ET technology can be a useful tool to evaluate reading of subjects while wearing PPLs with different power distribution maps and it can be used to validate the effectiveness of PPL designs optimized for specific visual tasks.

## Supporting information

S1 File (RAR)
