# Supplementary material for: Correlation between reading time and characteristics of eye fixations and progressive lens design
Source: PLoS One. 2023 Mar 27;18(3):e0281861. doi: 10.1371/journal.pone.0281861 (PMC10042365; doi:10.1371/journal.pone.0281861)
Supplement: Attachment — Submitted filename: Response to Reviewers.docx [file pone.0281861.s002.docx]

**PABLO CONCEPCIÓN GRANDE M.Sc.**

Indizen Optical Technologies,SL.

Calle suero de quiñones 34-36, 3º

28005 MADRID, Spain

[pconcepcion@iot.es](mailto:pconcepcion@iot.es)

Dec 7, 2022

Hereafter we provide replies to the points raised by the reviewers, and describe the changes made to the manuscript:

**Reviewer #1: Manuscript Number PONE-D-22-19980**

**1. IS THE MANUSCRIPT TECHNICALLY SOUND, AND DO THE DATA SUPPORT THE CONCLUSIONS? PARTLY

Design for Visual Tasks:**
**- The authors have measured the reading tasks at distance with a straight-ahead position, and two off-axis positions with the head turned 10deg and 15deg relative to the target [Lines 147-154]. Outcome variables of total reading time, total fixation time, and number of fixations were obtained. A derived variable of average fixation duration was also computed. Although the outcome variables were measured at the different orientations there were no data presented for the different orientations.**

**- In the results section, it appears that the outcome variables are reported as an average of the three positions with each lens – I was, at first, not clear why. The best description for why appears in the penultimate paragraph of the conclusion ([L257-263]) and I would suggest that this text is moved to the methods. Their analysis says “Nine text blocks for the different experimental conditions (3 PPLs and 3 rotations) were used in a randomized order.” [Line 151-152] and were analyzed using “…a randomized complete block statistical test.” [Line 178]; statistically I would interpret this as the angle of rotation being treated as a nuisance variable – I’m not sure if this was the author’s intention? Did the authors consider treating the angle of orientation as a dependent variable, from which the outcome variables might be described by an interaction between design and orientation relative to the target? This would give some validation for both the experimental design and the impact of peripheral lens areas on performance. Even if it is not included in the statistical analysis, descriptive results would be beneficial to their manuscript.**

According to the reviewer comments, an explanation about the necessity to rotate participants was added in the methods section as follows:

“Distance-reading performance was recorded while reading a text shown on a screen monitor (Asus LCD Monitor VP228HE 21.5’’) located at 5.25m. The text subtended 4.2° horizontally and 2.3° vertically and was composed of 5 text lines having a font size of 0.4logMAR. To evaluate a greater area, subjects were rotated into 3 different orientations: on-axis and two off-axis reading positions (10° and 15°). Off-axis rotations were induced using a rotation platform with a chinrest to prevent head mobility (Fig 3). For each position, subjects were asked to read out loud the text displayed on the screen with the 3 PPLs. Therefore, nine reading texts for the different experimental conditions (3 PPLs and 3 rotations) were used in a randomized order.

Near-reading performance was assessed at 0.37m using 3 different reading texts (randomized for each PPL) displayed on a screen (Microsoft Surface PRO 4 12.3"). Each reading text was composed of three columns with five text lines each with a font size of 0.4logMAR and a viewing angle of 28° horizontally and 3.6° vertically. A table with a chinrest was used to prevent head movements and ensure subjects used the PPL central and lateral regions.”

Following the reviewer's recommendations, the statistical analysis has been improved. The analysis has been carried out considering the different orientations of the text for distance and near vision. As expected, there are differences between the central and peripheral regions of the lens. This result has been added in the result section of the manuscript as follows:

Results section has been improved as follows:

“**Distance-reading vision**: Statistically significant differences were found between PPLs for distance-reading vision. When subjects were using a PPL optimized for distance vision, reading time; total duration of fixations and number of fixations decreased in comparison with PPL-Balance and PPL-Near. Statistically significant higher reading time and number of fixations were found for off-axis orientations in comparison with on-axis (Fig 5 and table 2).

Fig 5. Variations in reading time, fixation time and fixation count depending on the PPL (A), the orientation (B) and the interactions of PPL and orientation (C) for distance-reading task.

Table 2. Detailed statistics for Fig 5.

|  | **ANOVA test for lens design** | | | | **LSD Comparisons for lens design** | | |
| --- | --- | --- | --- | --- | --- | --- | --- |
|  | Df | Mean square | F-ratio | P-value | Balance/Near  (Diff limit) | Balance/Distance  (Diff+/-limit) | Distance/Near  (Diff+/-limit) |
| Reading time | 2 | 39.31 | 8.89 | <0.001* | -0.49*+/-*0.64 | 0.86*+/-*0.64* | -1.35*+/-*0.64* |
| Fixation time | 2 | 31.62 | 6.45 | 0.002* | -0.74*+/-*0.68* | 0.48*+/-*0.68 | -1.22*+/-*0.68* |
| Fixation count | 2 | 333.68 | 6.16 | 0.003* | -0.93*+/-*2.25 | 2.89*+/-*2.25* | -3.8*+/-*2.25* |
|  | **ANOVA test for orientation** | | | | **LSD comparisons for orientation** | | |
|  | Df | Mean square | F-ratio | P-value | On-axis/10º off-axis  (Diff+/-limit) | On-axis/15º off-axis  (Diff+/-limit) | 10 º/15 º off-axis  (Diff+/-limit) |
| Reading time | 2 | 21.47 | 4.86 | 0.01* | -0.69*+/-*0.64* | -0.98*+/-*0.64* | -0.29*+/-*0.64 |
| Fixation time | 2 | 0.39 | 0.08 | 0.924 | -0.13*+/-*0.68 | -0.09*+/-*0.68 | 0.04*+/-*0.68 |
| Fixation count | 2 | 1193.90 | 22.03 | <0.001* | -3.34*+/-*2.25* | -7.52*+/-*2.25* | -4.19*+/-*2.25* |

Multifactorial ANOVA test with pos-hoc comparisons using LSD. * Shows significance at the 0.05 level.

**Near-reading vision:** Statistically significant differences were found between PPLs for near-reading vision. When subjects were using a PPL optimized for near vision, reading time, total duration of fixations and the number of fixations decreased in comparison with PPL-Balance and PPL-Distance. Statistically significant lower reading time, lower fixation time and less fixation count were found for central column in comparison with peripheral columns (Fig 6 and table 3).

Fig 6. Variations in reading time, fixation time and fixation count depending on the PPL (A), the orientation (B) and the interactions of PPL and orientation (C) for near-reading task.

Table 3. Detailed statistics for Fig 6.

|  | **ANOVA test for lens design** | | | | **LSD Comparisons for lens design** | | |
| --- | --- | --- | --- | --- | --- | --- | --- |
|  | Df | Mean squeare | F-ratio | P-value | Balance/Near  (Diff+/-limit) | Balance/Distance  (Diff+/-limit) | Distance/Near  (Diff+/-limit) |
| Reading time | 2 | 135.30 | 12.24 | <0.001* | 1.64+/-0.81* | -0.86+/-0.81* | 2.50+/-0.81* |
| Fixation time | 2 | 54.27 | 7.79 | <0.001* | 0.97+/-0.58* | -0.63+/-0.58* | 1.60+/-0.58* |
| Fixation count | 2 | 1127.08 | 19.12 | <0.001* | 4.40+/-2.36* | -2.88+/-2.36* | 7.27+/-2.36* |
|  | **ANOVA test for reading columns** | | | | **LSD Comparisons for reading columns** | | |
|  | Df | Mean squeare | F-ratio | P-value | Center/Dominant  (Diff+/-limit) | Center/ Non-dominant  (Diff+/-limit) | Dominant/Non- dominant  (Diff+/-limit) |
| Reading time | 2 | 90.37 | 8.18 | <0.001* | -2.05+/-0.81* | -1.28+/-0.81* | 0.77+/-0.81 |
| Fixation time | 2 | 13.67 | 1.96 | 0.143 | -0.70+/-0.58* | -0.70+/-0.58* | 0.01+/-0.80 |
| Fixation count | 2 | 567.62 | 9.63 | <0.001* | -5.18+/-2.36* | -2.99+/-2.36* | 2.19+/-2.36 |

Multifactorial ANOVA test with pos-hoc comparisons using LSD. * Shows significance at the 0.05 level.

**- For the near reading task the head position was not tilted relative to the text (“3 different text blocks (randomized for each PPL) displayed on a screen” [Line 157]). It became clearer here that the authors are using ‘blocks’ to describe experimental units, and the statistical analysis run appears to be a repeated measures ANOVA with PPL design as a factor. If this is the case, then it should be included in the description of the analysis.**

In response to the reviewer’s comment, the Statistical analysis section has been modified to be clearer as follows:

“*Statistical Analysis: Statgraphics Centurion XVI.II Software was used in all the statistical analyses carried out in this research. Differences in eye movements depending on the power distribution maps of PPLs and orientations for distance and columns for near-reading vision were analyzed using a multifactorial ANOVA test, the statistical power was set at 0.8 and the level of significance at 0.05. To find differences in which means are significantly different from each other, a LSD post-hoc test was performed. Analyzed variables were reading time, fixation time, and fixation count*.”

In addition, to clarify the expression “text block”, it has been replaced by "reading text” throughout the manuscript as in the following example:

“Near-reading performance was assessed at 0.37m using 3 different reading texts (randomized for each PPL) displayed on a screen (Microsoft Surface PRO 4 12.3"). Each reading text was composed of three columns with five text lines each with a font size of 0.4logMAR and a viewing angle of 28° horizontally and 3.6° vertically. A table with a chinrest was used to prevent head movements and ensure subjects used the PPL central and lateral regions (Fig 4).”

**Conclusions & Supporting Data**

According to all reviewer comments and suggestions, the results section and therefore the statistical analysis has been improved as follows:

Results section has been improved as follows:

“**Distance-reading vision**: Statistically significant differences were found between PPLs for distance-reading vision. When subjects were using a PPL optimized for distance vision, reading time; total duration of fixations and number of fixations decreased in comparison with PPL-Balance and PPL-Near . Statistically significant higher reading time and number of fixations were found for off-axis orientations in comparison with on-axis (Fig 5and table 2).

Fig 5. Variations in reading time, fixation time and fixation count depending on the PPL (A), the orientation (B) and the interactions of PPL and orientation (C) for distance-reading task.

Table 2. Detailed statistics for Fig 5.

|  | **ANOVA test for lens design** | | | | **LSD Comparisons for lens design** | | |
| --- | --- | --- | --- | --- | --- | --- | --- |
|  | Df | Mean square | F-ratio | P-value | Balance/Near  (Diff limit) | Balance/Distance  (Diff+/-limit) | Distance/Near  (Diff+/-limit) |
| Reading time | 2 | 39.31 | 8.89 | <0.001* | -0.49*+/-*0.64 | 0.86*+/-*0.64* | -1.35*+/-*0.64* |
| Fixation time | 2 | 31.62 | 6.45 | 0.002* | -0.74*+/-*0.68* | 0.48*+/-*0.68 | -1.22*+/-*0.68* |
| Fixation count | 2 | 333.68 | 6.16 | 0.003* | -0.93*+/-*2.25 | 2.89*+/-*2.25* | -3.8*+/-*2.25* |
|  | **ANOVA test for orientation** | | | | **LSD comparisons for orientation** | | |
|  | Df | Mean square | F-ratio | P-value | On-axis/10º off-axis  (Diff+/-limit) | On-axis/15º off-axis  (Diff+/-limit) | 10 º/15 º off-axis  (Diff+/-limit) |
| Reading time | 2 | 21.47 | 4.86 | 0.01* | -0.69*+/-*0.64* | -0.98*+/-*0.64* | -0.29*+/-*0.64 |
| Fixation time | 2 | 0.39 | 0.08 | 0.924 | -0.13*+/-*0.68 | -0.09*+/-*0.68 | 0.04*+/-*0.68 |
| Fixation count | 2 | 1193.90 | 22.03 | <0.001* | -3.34*+/-*2.25* | -7.52*+/-*2.25* | -4.19*+/-*2.25* |

Multifactorial ANOVA test with pos-hoc comparisons using LSD. * Shows significance at the 0.05 level.

**Near-reading vision:** Statistically significant differences were found between PPLs for near-reading vision. When subjects were using a PPL optimized for near vision, reading time, total duration of fixations and the number of fixations decreased in comparison with PPL-Balance and PPL-Distance. Statistically significant lower reading time, lower fixation time and less fixation count were found for central column in comparison with peripheral columns (Fig 6 and table 3).

Fig 6. Variations in reading time, fixation time and fixation count depending on the PPL (A), the orientation (B) and the interactions of PPL and orientation (C) for near-reading task.

Table 3. Detailed statistics for Fig 6.

|  | **ANOVA test for lens design** | | | | **LSD Comparisons for lens design** | | |
| --- | --- | --- | --- | --- | --- | --- | --- |
|  | Df | Mean squeare | F-ratio | P-value | Balance/Near  (Diff+/-limit) | Balance/Distance  (Diff+/-limit) | Distance/Near  (Diff+/-limit) |
| Reading time | 2 | 135.30 | 12.24 | <0.001* | 1.64+/-0.81* | -0.86+/-0.81* | 2.50+/-0.81* |
| Fixation time | 2 | 54.27 | 7.79 | <0.001* | 0.97+/-0.58* | -0.63+/-0.58* | 1.60+/-0.58* |
| Fixation count | 2 | 1127.08 | 19.12 | <0.001* | 4.40+/-2.36* | -2.88+/-2.36* | 7.27+/-2.36* |
|  | **ANOVA test for reading columns** | | | | **LSD Comparisons for reading columns** | | |
|  | Df | Mean squeare | F-ratio | P-value | Center/Dominant  (Diff+/-limit) | Center/ Non-dominant  (Diff+/-limit) | Dominant/Non- dominant  (Diff+/-limit) |
| Reading time | 2 | 90.37 | 8.18 | <0.001* | -2.05+/-0.81* | -1.28+/-0.81* | 0.77+/-0.81 |
| Fixation time | 2 | 13.67 | 1.96 | 0.143 | -0.70+/-0.58* | -0.70+/-0.58* | 0.01+/-0.80 |
| Fixation count | 2 | 567.62 | 9.63 | <0.001* | -5.18+/-2.36* | -2.99+/-2.36* | 2.19+/-2.36 |

Multifactorial ANOVA test with pos-hoc comparisons using LSD. * Shows significance at the 0.05 level.

**- 1)The main conclusion that “…reading time and characteristics of fixations of PPL users are affected by the power distribution of the PPL…” [L:201-202] is quite general (and is supported, in general) but is a little bit superficial. 2)More specifically, their study shows a difference between the distance and near PPL designs for some of the outcome variables, but neither differ from the balance PPL design in the majority of cases – which is not identified in the manuscript.**

After reviewing the manuscript, the results section has been rewritten answering the reviewers comments and following their suggestions improving the statistical analysis, thus the main conclusion has been modified to be more precise as follows:

“The results of this study show that when participants are induced to look through different areas of the lens, there are variations in reading time, total duration of fixations, and fixation count depending on the visual area of the PPL. A PPL design with a wider distance visual area provides lower reading time and less fixation count than PPL-Balance and PPL-Near, improving reading at distance vision. Similarly for near-vision reading, a PPL design with a wider near area provides lower reading time, lower total fixation time, and less fixation count than PPL-Balance and PPL-Distance, improving reading at near vision. Also, it has been observed the influence of peripheral distortions of the PPLs in reading performance. More specifically, reading time and fixation count was higher at off-axis positions in comparison with on-axis positions both at near and distance reading tasks.”**- The magnitude of astigmatism was not a variable in the model - other than being encompassed, very generally, by the design variable. It’s a little speculative to attribute the difference in performance entirely to unwanted astigmatism in the designs, rather than other reasons not reported such as the impact of the refractive error of the sample or the previous PPL design experience of the subjects. Not all subjects show exactly the same pattern of responses.**

In response to the reviewer, previous studies had studied the influence of unwanted astigmatism in eye movements, but in the current work we have analyzed the influence of the PPLs’ fields of view for distance and near vision on reading time and fixations characteristics. Therefore, following the reviewer comment we have modified the following paragraph of the discussion using visual field instead of astigmatism as follows:

“In accordance with them, our study shows that when visual fields are constrained, the reading time, complete fixation time and fixation count increase. In addition, our results show that when using the central area of the lens, with less distortion than the periphery, reading time, complete fixation time and fixation count decrease”

Regarding unwanted astigmatism influence, in this study we have studied the influence of fields of view on reading performance and not only the effect of unwanted astigmatism. For this reason, fields of view at distance and near area have been analyzed according to Sheedy et al. criteria as follows:

|  | **Sheedy's area** | | | | | | **Difference compared to balance** | | | |
| --- | --- | --- | --- | --- | --- | --- | --- | --- | --- | --- |
|  | **PPL-Balance** | | **PPL-Distance** | | **PPL-Near** | | **Distance area (%)** | | **Near area (%)** | |
| ID | **Distance area(mm^2^)** | **Near area(mm^2^)** | **Distance area(mm^2^)** | **Near area(mm^2^)** | **Distance area(mm^2^)** | **Near area(mm^2^)** | **PPL-Distance** | **PPL-Near** | **PPL-Distance** | **PPL-Near** |
| 102 | 38,51 | 43,18 | 45,26 | 36,7 | 30,86 | 56,59 | 18% | -20% | -15% | 31% |
| 149 | 38,41 | 35,08 | 53,28 | 31,6 | 30,635 | 46,29 | 39% | -20% | -10% | 32% |
| 197 | 37,835 | 37,36 | 47,76 | 32,51 | 29,81 | 48,21 | 26% | -21% | -13% | 29% |
| 546 | 39,435 | 34,98 | 53,52 | 31,32 | 30,415 | 43,99 | 36% | -23% | -10% | 26% |
| 687 | 36,3 | 40,95 | 42,79 | 32,68 | 29,2 | 53,47 | 18% | -20% | -20% | 31% |
| 771 | 37,52 | 34,1 | 50,06 | 30,05 | 28,905 | 43,63 | 33% | -23% | -12% | 28% |
| 880 | 38,97 | 35,08 | 51,41 | 31,01 | 30,17 | 44,26 | 32% | -23% | -12% | 26% |
| 899 | 38,345 | 35,27 | 50,71 | 31,95 | 29,67 | 44,35 | 32% | -23% | -9% | 26% |
| 972 | 38,485 | 35,5 | 48,7 | 31,44 | 29,905 | 45,19 | 27% | -22% | -11% | 27% |
| 1692 | 38,04 | 35,37 | 49,24 | 31,06 | 29,37 | 44,99 | 29% | -23% | -12% | 27% |
| 1779 | 39,035 | 42,42 | 47,32 | 38,25 | 31,015 | 53,68 | 21% | -21% | -10% | 27% |
| 2200 | 39,23 | 39,49 | 47,035 | 35,9 | 31,035 | 50,53 | 20% | -21% | -9% | 28% |
| 2384 | 37,68 | 41,16 | 46,305 | 36,11 | 30,13 | 51,71 | 23% | -20% | -12% | 26% |
| 2404 | 38,625 | 35,07 | 51,11 | 31,06 | 29,96 | 44,2 | 32% | -22% | -11% | 26% |
| 2575 | 37,66 | 38,31 | 47,445 | 32,36 | 29,93 | 49,36 | 26% | -21% | -16% | 29% |
| 2855 | 36,455 | 32,29 | 47,305 | 28,51 | 28,34 | 41,26 | 30% | -22% | -12% | 28% |
| 2859 | 39,53 | 35,76 | 53,995 | 31,98 | 30,325 | 44,66 | 37% | -23% | -11% | 25% |
| 2865 | 38,755 | 35,7 | 50,545 | 32,16 | 30,03 | 45,09 | 30% | -23% | -10% | 26% |
| 2873 | 37,18 | 33,1 | 48,755 | 29,59 | 28,71 | 42,48 | 31% | -23% | -11% | 28% |
| 2916 | 39,09 | 41 | 49,98 | 37,25 | 30,32 | 52,19 | 28% | -22% | -9% | 27% |
| 2923 | 37,985 | 37,71 | 48,415 | 33,42 | 29,725 | 48,1 | 27% | -22% | -11% | 28% |
| 2943 | 38,21 | 36,58 | 49,35 | 33,39 | 29,845 | 46,18 | 29% | -22% | -9% | 26% |
| 2951 | 39,03 | 35,31 | 49,14 | 30,2 | 30,51 | 46,22 | 26% | -22% | -14% | 31% |
| 2954 | 38,685 | 38 | 48,085 | 33,1 | 30,345 | 48,07 | 24% | -22% | -13% | 26% |
| 2999 | 38,3 | 34,35 | 51,005 | 30,64 | 29,555 | 43,02 | 33% | -23% | -11% | 25% |
| 3037 | 37,375 | 33,86 | 48,255 | 30,18 | 29,02 | 43,04 | 29% | -22% | -11% | 27% |
| 3085 | 37,755 | 36,29 | 49,03 | 33,03 | 29,415 | 45,78 | 30% | -22% | -9% | 26% |
| 3088 | 40 | 37,61 | 53,175 | 33,57 | 31,18 | 47,03 | 33% | -22% | -11% | 25% |

As can be seen in the table above, the difference in visual areas for each participant remains relatively stable. PPL-Distance has a 29±5% wider field of view and the PPL-Near lens a 22±1% narrower field of view in the distance zone compared to the control lens (PPL-Balance). In the near zone, compared to the control lens, the PPL-Near has a 27±2% wider field of view and the PPL-Distance a 12±2% narrower field of view. This has been included in method section as follows:

**“Progressive Power Lenses:** Three free-form PPL designs individualized with different power distribution maps were developed ad-hoc for this research: 1) PPL-Balance, the control lens (Endless Steady Balance, IOT, Madrid, Spain), 2) PPL-Distance, a lens with a wider visual area for distance vision (Endless Steady Distance, IOT, Madrid, Spain) and 3) PPL-Near, a lens with wider visual area for near vision (Endless Steady Near, IOT, Madrid, Spain). Mean power, cylinder power maps distribution, Sheedy contours and visual areas according to Sheedy’s criteria (28) for a plano addition 2.00D prescription using standard parameters are shown in Fig 1.

Lenses were mounted in a specific clip-on (developed ad-hoc) that was attached to the front of the ET eyeglasses. This set up allowed the direct recording of the pupils without any interference from the progressive lenses. (Fig 2).

Fig 1: Mean power, cylinder power maps distribution, and visual areas according to Sheedy’s criteria for plano addition 2.00D prescription using standard parameters of the PPLs tested in the research A) PPL-Balance. B) PPL-Distance. C) PPL-Near

Fig 2: Picture of the metallic frame developed ad-hoc for this study attached to Tobii Glasses PRO 3.

Lenses were calculated using fitting parameters and position of wear of the ET with the clip-on using advanced optical design software (Freeform designer, IOT, Madrid, Spain) to reduce oblique aberrations and maintain fields of view stable independent of each subject's prescription and addition power for all gaze directions. According to Sheddy's criteria, the PPL-Distance lens has a 29% wider field of view and the PPL-Near lens a 22% narrower field of view in the distance zone compared to the control lens (PPL-Balance). In the near zone, compared to the control lens, the PPL-Near has a 27% wider field of view and the PPL-Distance a 12% narrower field of view”

**- I was a little bit surprised by the level of astigmatism in the peripheral zones of the lenses (for the plano +2.00 Add example; Fig 1), because the areas with ~1.75 or more astigmatism looks larger than I would have expected. I’m not sure if this arises because of the need to create a lens to go over the eye-tracker equipment, as the authors do say that the lenses are ‘…freeform…’ [L126] and are optimised for the position of wear parameters [L120-121]?**

Responding reviewer’s comments, Concerning Free-form technology, and optimization: When light rays from an object enter a lens at an angle, such as when a patient uses the lens' peripheral area to look away from the optical center or when the lenses are not perfectly perpendicular to the patient's viewing direction, oblique aberrations result. In these cases, the lens is essentially giving a prescription with the sphere, cylinder, and axis values set incorrectly, resulting in distorted vision and a reduction in the visual field. Recently, the process of making lenses has been improved with the irruption of free-form technology. Free-form technology enables the creation of arbitrary surfaces as opposed to conventional manufacturing procedures, which are limited to producing a sphere or torus on the back surface of the lens. Therefore, a much better compensation of the oblique aberrations is possible when spherical molded blanks are combined with a back surface that is manufactured point by point. Using advanced optical design software, it is possible to optimize the back lens surface to correct for oblique aberrations while considering the patient's position of wear (interpupillary distance, fitting height, back vertex distance, pantoscopic angle, wrapping angle, and near working distance), the lens' refractive index, prescription, base curve, and frame characteristics. The outcome is a user-compensated lens with a unique back surface customized for each patient's wearing position(1–3). So, this customization means that the design remains relatively stable and that there is no variation in the fields of view with the prescription and/or addition of each subject(4).

To clarify this point, the following paragraph has been included in method section:

*“Lenses were calculated using fitting parameters and position of wear using advanced optical design software (Freeform designer, IOT, Madrid, Spain) to reduce oblique aberrations and maintain fields of view stable independent of each subject's prescription and addition power for all gaze directions. According to Sheedy’s criteria, the PPL-Distance lens has a 29% wider field of view and the PPL-Near lens a 22% narrower field of view in the distance zone compared to the control lens (PPL-Balance). In the near zone, compared to the control lens, the PPL-Near has a 27% wider field of view and the PPL-Distance a 12% narrower field of view”*

**- There’s a portion of the discussion that refers to other research where the authors state “Changes during reading observed between different types of PPLs are similar to the differences found between good and poor readers.” [L 239-240]. This is correct in as much as the direction is the same – that is, the reading time is longer and the number of fixations are more numerous with the Distance PPL for the reading task than with the Near PPL. However, as a reader you come away with the sense that the magnitude of the difference is similar – i.e. you are a ‘poor’ reader with a Distance PPL compared with being a ‘good’ reader with a Near PPL. This would be an incorrect inference and is unclear for the reader. Bucci et al (for example)** **report a 2.6x increase in the number of fixations for dyslexic (‘poor’) versus non-dyslexic readers (‘good’) and ~2x fixation duration, whereas it is a 1.2x difference with a Distance PPL versus a Near PPL.**

Following reviewer comments, the paragraph between lines 239 and 255 has been modified to be more correct and precise as follows:

“There are some studies where reading pattern has been evaluated between good and poor readers. Bucci et al (10) studied how fixations are affected during reading on 12 dyslexic and 19 non dyslexic children using an ET with a sample rate of 300Hz. Subjects were asked to read aloud a text composed by 4 lines located at 58 cm. Results showed that dyslexic children had more and longer fixations than non-dyslexic children. Griffin et al (15) compared fixation count and reading time of 13 poor readers and 13 good readers of an age between 9 and 11 years old using a video camera. The analysis showed that poor readers needed more fixations per word and more time than those participants with normal reading skills. A similar result was obtained by Landerl et al (34) using an Eye Link 1000 ET on 137 third and fourth graders classified as good or poor readers. Results showed that poor readers had higher number of fixations and total fixation time than those participants classified as good readers. Poor readers need more and longer fixations than good readers, resulting in longer reading times. Our study shows differences between PPLs in the same variables than these studies, but in a different magnitude. Those PPL with a narrower field of view for the specific reading-task produce more and longer fixations, and thus higher reading time. This result suggests that there is a deterioration of reading due the power distribution of a PPL.”

**- [L237-238] “These results are consistent with our findings on reading skills being affected by the unwanted astigmatism…”. The results referred to would be consistent with unwanted astigmatism affecting ease of reading and supports the author’s conjecture that the unwanted astigmatism associated with the design is the culprit… but their experiment did not explicitly include astigmatism in the model, it included lens design; the astigmatism component is inferred.**

Following reviewer comment, this sentence has been modified to be more correct as follows:

*“These results are consistent with our findings on reading being affected by the field of view of the PPLs.”*

**- [L252-255] These sentences need additional clarity to indicate that the magnitude of impairment reported for ‘poor’ and ‘good’ readers is not the same as reported in their study.**

Following reviewer comment, this point has been clarified as part of the discussion as follows:

“There are some studies where reading pattern has been evaluated between good and poor readers. Bucci et al (10) studied how fixations are affected during reading on 12 dyslexic and 19 non dyslexic children using an ET with a sample rate of 300Hz. Subjects were asked to read aloud a text composed by 4 lines located at 58 cm. Results showed that dyslexic children had more and longer fixations than non-dyslexic children. Griffin et al (15) compared fixation count and reading time of 13 poor readers and 13 good readers of an age between 9 and 11 years old using a video camera. The analysis showed that poor readers needed more fixations per word and more time than those participants with normal reading skills. A similar result was obtained by Landerl et al (34) using an Eye Link 1000 ET on 137 third and fourth graders classified as good or poor readers. Results showed that poor readers had higher number of fixations and total fixation time than those participants classified as good readers. Poor readers need more and longer fixations than good readers, resulting in longer reading times. Our study shows differences between PPLs in the same variables than these studies, but in a different magnitude. Those PPL with a narrower field of view for the specific reading-task produce more and longer fixations, and thus higher reading time. This result suggests that there is a deterioration of reading due the power distribution of a PPL.”

**2. HAS THE STATISTICAL ANALYSIS BEEN PERFORMED APPROPRIATELY AND RIGOROUSLY? NO

- Design of the PPL appears to be a main effect in their analysis. The authors report in the methods that “…PPL-Balance, the control lens …”. I was expecting, given its designation as a control lens, that the analysis might include contrasts between the PPL-Bal and the two lenses optimised for a specific distance (i.e. comparing the PPL-Distance design with the PPL-Balance and comparing the PPL-Near design with the PPL-Balance only).**

**- As it was not clear what analysis was run, I ran the data provided with a simple repeated measures ANOVA (with design as a factor) and got identical probability values as reported in Table 2 & Table 3. I was unable to confirm this was the same as the author’s analysis as neither F values or degrees of freedom were provided. Secondary to running the data, I noticed that many of the main effects have significant sphericity, so reporting of corrected probability values (Greenhouse-Geisser or Huynh-Feldt) is indicated. It does not change the significant/non-significant findings but is more precise.**

**- In the absence of contrasts, no post-hoc testing was reported on the main effect of PPL design, to indicate which designs yielded an average difference in performance. This may have been done (the authors report that [L188-189] “…was decreased in comparison with PPL-Balance and PPL-Near.”) but the probability values (or procedure) associated with the specific comparisons are not given. The omnibus probability value does not apply to the specific comparisons.**

**- Unless average fixation duration is a motivating variable of interest (it didn’t appear to be from the methods or the discussion), I don’t see the value of performing statistical testing on total fixation duration, fixation counts and average fixation duration (derived from fixation duration/fixation count). Shouldn’t it be total reading time and average fixation duration or total reading time, total fixation time, number of fixations? It is of value to see the data for average fixation duration, but I don’t think they have the experimental power (or the experimental intention) to run 3 separate ANOVAs on the same data.**

In response to the reviewer's comments, a post-hoc analysis has been carried out in which it has been verified that the PPL-Distance design has a different behavior to the control lens (PPL-Balance) in the distance test in all the variables analyzed. Likewise, this analysis shows that the PPL-Near design performs differently from the control lens (PPL-Balance) in all the variables analyzed for the near test. Following the reviewer's suggestions, the type of statistical test performed (multifactorial ANOVA) and its F-values and degrees of freedom as well as the contrasts analysis (LSD test) have been included in the manuscript. Following the reviewer's recommendations, the average fixation duration has been removed from the manuscript.

Results section has been improved as follows:

“**Distance-reading vision**: Statistically significant differences were found between PPLs for distance-reading vision. When subjects were using a PPL optimized for distance vision, reading time; total duration of fixations and number of fixations decreased in comparison with PPL-Balance and PPL-Near. Statistically significant higher reading time and number of fixations were found for off-axis orientations in comparison with on-axis (Fig 5 and table 2).

Fig 5. Variations in reading time, fixation time and fixation count depending on the PPL (A), the orientation (B) and the interactions of PPL and orientation (C) for distance-reading task.

Table 2. Detailed statistics for Fig 5.

|  | **ANOVA test for lens design** | | | | **LSD Comparisons for lens design** | | |
| --- | --- | --- | --- | --- | --- | --- | --- |
|  | Df | Mean square | F-ratio | P-value | Balance/Near  (Diff limit) | Balance/Distance  (Diff+/-limit) | Distance/Near  (Diff+/-limit) |
| Reading time | 2 | 39.31 | 8.89 | <0.001* | -0.49*+/-*0.64 | 0.86*+/-*0.64* | -1.35*+/-*0.64* |
| Fixation time | 2 | 31.62 | 6.45 | 0.002* | -0.74*+/-*0.68* | 0.48*+/-*0.68 | -1.22*+/-*0.68* |
| Fixation count | 2 | 333.68 | 6.16 | 0.003* | -0.93*+/-*2.25 | 2.89*+/-*2.25* | -3.8*+/-*2.25* |
|  | **ANOVA test for orientation** | | | | **LSD comparisons for orientation** | | |
|  | Df | Mean square | F-ratio | P-value | On-axis/10º off-axis  (Diff+/-limit) | On-axis/15º off-axis  (Diff+/-limit) | 10 º/15 º off-axis  (Diff+/-limit) |
| Reading time | 2 | 21.47 | 4.86 | 0.01* | -0.69*+/-*0.64* | -0.98*+/-*0.64* | -0.29*+/-*0.64 |
| Fixation time | 2 | 0.39 | 0.08 | 0.924 | -0.13*+/-*0.68 | -0.09*+/-*0.68 | 0.04*+/-*0.68 |
| Fixation count | 2 | 1193.90 | 22.03 | <0.001* | -3.34*+/-*2.25* | -7.52*+/-*2.25* | -4.19*+/-*2.25* |

Multifactorial ANOVA test with pos-hoc comparisons using LSD. * Shows significance at the 0.05 level.

**Near-reading vision:** Statistically significant differences were found between PPLs for near-reading vision. When subjects were using a PPL optimized for near vision, reading time, total duration of fixations and the number of fixations decreased in comparison with PPL-Balance and PPL-Distance. Statistically significant lower reading time, lower fixation time and less fixation count were found for central column in comparison with peripheral columns (Fig 6 and table 3).

Fig 6. Variations in reading time, fixation time and fixation count depending on the PPL (A), the orientation (B) and the interactions of PPL and orientation (C) for near-reading task.

Table 3. Detailed statistics for Fig 3.

|  | **ANOVA test for lens design** | | | | **LSD Comparisons for lens design** | | |
| --- | --- | --- | --- | --- | --- | --- | --- |
|  | Df | Mean squeare | F-ratio | P-value | Balance/Near  (Diff+/-limit) | Balance/Distance  (Diff+/-limit) | Distance/Near  (Diff+/-limit) |
| Reading time | 2 | 135.30 | 12.24 | <0.001* | 1.64+/-0.81* | -0.86+/-0.81* | 2.50+/-0.81* |
| Fixation time | 2 | 54.27 | 7.79 | <0.001* | 0.97+/-0.58* | -0.63+/-0.58* | 1.60+/-0.58* |
| Fixation count | 2 | 1127.08 | 19.12 | <0.001* | 4.40+/-2.36* | -2.88+/-2.36* | 7.27+/-2.36* |
|  | **ANOVA test for reading columns** | | | | **LSD Comparisons for reading columns** | | |
|  | Df | Mean squeare | F-ratio | P-value | Center/Dominant  (Diff+/-limit) | Center/ Non-dominant  (Diff+/-limit) | Dominant/Non- dominant  (Diff+/-limit) |
| Reading time | 2 | 90.37 | 8.18 | <0.001* | -2.05+/-0.81* | -1.28+/-0.81* | 0.77+/-0.81 |
| Fixation time | 2 | 13.67 | 1.96 | 0.143 | -0.70+/-0.58* | -0.70+/-0.58* | 0.01+/-0.80 |
| Fixation count | 2 | 567.62 | 9.63 | <0.001* | -5.18+/-2.36* | -2.99+/-2.36* | 2.19+/-2.36 |

Multifactorial ANOVA test with pos-hoc comparisons using LSD. * Shows significance at the 0.05 level.

**3. HAVE THE AUTHORS MADE ALL DATA UNDERLYING THE FINDINGS IN THEIR MANUSCRIPT FULLY AVAILABLE? NO**

**- The authors give their inclusion/exclusion criteria, but do not give the reader a comprehensive description of (a) the refractive errors for their sample, or (b) the general design characteristics (i.e balanced, distance or near PPL) worn by their subjects for at least 6 months prior to the study.**

**- (a) Although the authors provide a representative power map for Plano with +2.00D Add for each lens design, I didn’t have a sense of how representative this was for the study sample. The authors do report the number of subjects with myopia/ hyperopia/ emmetropia and the range of Add powers [L 182-185], but do not indicate these characteristics by subject in their dataset. In general, PPLs will show a reduction in area and increased astigmatic blur with increasing Add power and the areas of clear vision may also be affected by the distance refractive error. Given that the discussion relies heavily on the impact of astigmatism from the less optimal areas of the lens, this seems to be relevant data to support their conclusions.**

**- (b) This might give some more practical insight into any observed changes in duration/number of fixations. For example, were the subjects that showed much longer times for the distance reading tasks with the distance or near PPL, those that were wearing a balance PPL prior to the study? Or were the longer times associated with subjects that had higher amounts of refractive cylinder? Or higher addition powers? Etc**

a) In response to this comment, a better description of refractive errors and addition of the sample was included in the results section as follows:

In response to the reviewer comments and as previously discussed in section “conclusions and supporting data” of this document, it has been shown theoretically that visual field of PPLs are not affected by the prescription or addition power of participants and has been included in the manuscript in methods section as follows:

**“Progressive Power Lenses:** Three free-form PPL designs individualized with different power distribution maps were developed ad-hoc for this research: 1) PPL-Balance, the control lens (Endless Steady Balance, IOT, Madrid, Spain), 2) PPL-Distance, a lens with a wider visual area for distance vision (Endless Steady Distance, IOT, Madrid, Spain) and 3) PPL-Near, a lens with wider visual area for near vision (Endless Steady Near, IOT, Madrid, Spain). Mean power, cylinder power maps distribution, Sheedy contours and visual areas according to Sheedy’s criteria (28) for a plano addition 2.00D prescription using standard parameters are shown in Fig 1.

Lenses were mounted in a specific clip-on (developed ad-hoc) that was attached to the front of the ET eyeglasses. This set up allowed the direct recording of the pupils without any interference from the progressive lenses. (Fig 2).

Fig 1: Mean power, cylinder power maps distribution, and visual areas according to Sheedy’s criteria for plano addition 2.00D prescription using standard parameters of the PPLs tested in the research A) PPL-Balance. B) PPL-Distance. C) PPL-Near

Fig 2: Picture of the metallic frame developed ad-hoc for this study attached to Tobii Glasses PRO 3.

Lenses were calculated using fitting parameters and position of wear of the ET with the clip-on using advanced optical design software (Freeform designer, IOT, Madrid, Spain) to reduce oblique aberrations and maintain fields of view stable independent of each subject's prescription and addition power for all gaze directions. According to Sheddy's criteria, the PPL-Distance lens has a 29% wider field of view and the PPL-Near lens a 22% narrower field of view in the distance zone compared to the control lens (PPL-Balance). In the near zone, compared to the control lens, the PPL-Near has a 27% wider field of view and the PPL-Distance a 12% narrower field of view”

Regarding refractive error, age, and gender distribution for each participant has been included in the dataset. In addition, although the sample size is very small, we have analyzed if there is a relationship between prescription or addition in the data obtained in this experiment, an analysis of correlations with the variables to be studied was carried out, but as expected no relevant data were obtained. The results obtained are shown below:

Near-reading analysis:

|  |  |
| --- | --- |
|  |  |
|  |  |

Distance-reading analysis:

|  |  |
| --- | --- |
|  |  |
|  |  |

b) Regarding the previous design they had before the experiment, even though the previous lens provider can be registered it is difficult to infer design characteristics of the wearers due to the huge variability of designs and calculation methods available in the market. The following paragraph was added to the section as follows:

“Finally, the previous PPL design used by each participant was not registered in our study due to the variability of options in the market. In future studies, it could be interesting to include this analysis to study the influence of adaptation to prior design in ocular movements using different PPL designs.”

**4. IS THE MANUSCRIPT PRESENTED IN AN INTELLIGIBLE FASHION AND WRITTEN IN STANDARD ENGLISH? YES**

**Specific typographical/grammatical suggestions:
[L64-67] [L82-86] Please move reference citation numbers to after the authors name, rather than the end of the sentences.**

Following reviewer comment, these sections of the manuscript was modified as follows**:**

“Garzia et al (16), simulated 2.00D of bilateral hyperopia on 19 adult subjects and reported an increase of 11% in reading time. Young and Hopkins et al (17,18), associated myopia with improvements in reading abilities. Wills et al (8) reported a reduction of reading speed by up to 24% when simulating astigmatism blur as low as 1.00D at 90°. A similar outcome was reported by Casagrande et al (19), who showed a reduction in reading speed by 35% with a simulated astigmatic blur of 0.75D at 90°”

“Several studies have used ET to analyze how eye movements during reading are affected by PPLs. The study of Han et al (24,25) used ET techniques to determine eye movements differences between PPLs and single-vision lenses. Concepcion et al (26) analyzed the differences in visual behavior of different visual areas of the PPLs during reading on a computer screen. Rifai et al (27) evaluated eye and head movements during driving in PPL users in comparison with non-PPL users. All of them showed that blur on the lateral regions of PPLs affects reading.”

**[L91-93] “The main objective of this study is to evaluate and compare reading time and characteristics of fixations when using three PPLs with different power distributions at distance-reading and near-reading tasks.” Suggest change to “… when performing a distance-reading and a near-reading task.”**

Responding reviewer comment, this has been changed as follows:

“The main objective of this study is to evaluate and compare reading time and characteristics of fixations when using three PPLs with different power distributions when performing a distance-reading and near-reading tasks”

**[L96-97] “…carried out to evaluate characteristics of fixations when reading…”. Suggest change to “…carried out to evaluate reading time and characteristics of fixations when reading…”.**

According to reviewer suggestion, this sentence has been changed as follows:

“A prospective observational longitudinal double-masked study was carried out to evaluate reading time and characteristics of fixations when reading with 3 different types of PPL.”

**[L99] Should the ethics approval number be included?**

In response to reviewer comment, this has been included:

“Full approval for the study was obtained from the Hospital Clínico San Carlos Ethics Committee’s (CEIC) Review Board (15/361-P).”

**[L100] Is it two pairs of eyeglasses? Or two lenses?**

In response to reviewer comment, this has been changed as follows:

“All subjects provided their written informed consent before starting the study and, at the end of the trial, subjects were compensated with two pair of eyeglasses.”

**[L102] “…and with previous experience of at least 6 months wearing PPLs.” Suggest change to “…and with at least 6 months prior experience of PPL wear.”**

In response to reviewer comment, this has been changed as follows:

“Participants: The study sample was comprised of presbyopic subjects of both genders aged over 45 and with at least 6 months prior experience of PPL wear.”

**[L106] “…binocular 4) Anisometropia…” is missing punctuation**

In response to reviewer comment, punctuation has been included:

“3) Best-corrected visual acuity (VA) better than 0.10logMAR monocular and 0.05logMAR binocular. 4) Anisometropia lower than 1.50D.”

**[L118] I believe ‘worth’ should be capitalized in “… worth test…”**

Following reviewer comment, this change has been made:

“, subjective refraction at distance and near vision, stereoacuity evaluation using Titmus test, cover test, Worth test, and ocular motility analysis.”

**[L125] “…between each measurement were done to avoid participants’ fatigue.”. Suggest ‘minimize’ be used instead of ‘avoid’.**

In response to reviewer comment, this has been changed as follows:

“Two-minute breaks between each measurement were done to minimize participants’ fatigue.”

**[L132] The power distribution maps are in Fig 1; Fig 2 is referenced in the text.**

In response to reviewer comment, this has been changed as follows:

“Mean power, cylinder power maps distribution, Sheedy contours and visual areas according to Sheedy’s criteria(4) for a plano addition 2.00D prescription using standard parameters are shown in Fig 1.”

**[L178] “…randomized complete block statistical test.” Suggest substituting repeated measures ANOVA for ‘statistical test’.**

**[L180] See the statistical analysis comments for the inclusion of the statistical analysis of average duration of fixations.**

In response to reviewer comments, statistical section has been changed as follows:

“Statistical Analysis: Statgraphics Centurion XVI.II Software was used in all the statistical analyses carried out in this research. Differences in eye movements depending on the power distribution maps of PPLs and orientations for distance and columns for near-reading vision were analyzed using a multifactorial ANOVA test, the statistical power was set at 0.8 and the level of significance at 0.05. To find differences in which means are significantly different from each other, a LSD post-hoc test was performed. Analyzed variables were reading time, fixation time, and fixation count.”

**[L185] “The addition of the participants…” Suggest changing to “The addition power for the participants…”**

According to reviewer comment, this has been changed on the manuscript as follows:

“The addition power of the participants ranged from 1D to 2.50D with and average value of 1.96±0.47 D.”

**[L204] “…improving reading skills…” Consider replacing skills (which implies more than reading speed/fixations) with a more representative word.**

Following reviewer comment, the term reading skills has been replaced by “reading” throughout the manuscript as in the following example:

“Rifai et al (9) evaluated eye and head movements during driving in PPL users in comparison with non-PPL users. All of them showed that blur on the lateral regions of PPLs affects reading.”

**[L211-212] “…subject’s midline at 0.6m, while their head was fixed with a chinrest.” Remove ‘while their head was fixed with a chinrest’ as the study analyzed eye and head movements without a chinrest.**

Following reviewer suggestion, this changed has been made in the manuscript:

“These results are in accordance with other studies that have used ET systems to evaluate the influence of PPLs in eye and head movements. In the study from Han et al(10), the differences between one single-vision lens and two PPLs on 11 presbyopic subjects was studied. Subjects had to read out loud a copy text printed in a standardized single page located along the subject’s midline at 0.60m. Eye and head positions were analyzed with the ISCAN integrated eye-head movement computer base system.”

**[L240] “…good and poor readers.. Bucci et al….” Remove extra period.**

Answering reviewer comment, the extra period was delated of this sentence:

“There are some studies where reading pattern has been evaluated between good and poor readers. Bucci et al”

**[L264] “…more natural condition it would…” Change condition to conditions.**

Following reviewer comment, this has been changed as follows:

“To simulate more natural conditions, it would be necessary to use a bigger screen with a wider visual field without the need of using a rotation platform.”

**[L265-267] “On the other hand, it would be interesting the evaluation of reading performance and ocular movements without the restrictions of using chinrest to avoid head movements.” Suggest changing to “****Similarly, it would be interesting to evaluate reading performance and ocular movements without the restriction of a chinrest.”**

Following reviewer suggestion, this sentence has been changed as follows:

To simulate more natural conditions, it would be necessary to use a bigger screen with a wider visual field without the need of using a rotation platform. Similarly, it would be interesting to evaluate reading performance and ocular movements without the restriction of a chinrest.

**[L269] “…with different power distributions at distance-reading…”. Suggest changing ‘at’ to ‘for’.**

Following reviewer comment, this has been changed as follows:

To conclude, reading time and characteristics of fixations change between PPLs with different power distributions for distance-reading and near-reading tasks. Thus, ET technology can be a useful tool to evaluate reading of subjects while wearing PPLs with different power distribution maps and it can be used to validate the effectiveness of PPL designs optimized for specific visual tasks.

**The references, in general, need some work. They are inconsistent with each other – for example: some have full journal titles, some abbreviated; some have year only, others have year and month, and others have year, month and day. Reference #s 4, 5, 7, 8, 9, 12, 16, 17, 21, 22, and 23 appear to be incomplete.**

Following the reviewer's comment, all references have been checked and corrected.

**Reviewer #2:**

**The manuscript describes a study using eye tracking to compare visual performance (tested in a reading task) with different progressive lenses. Focus of the study is on comparing lens designs optimized either for more far or more near vision tasks. The topic is very interesting and might have interesting results for ophthalmologists, optics designers and vision scientists. Nevertheless, I see some problems with the data analysis as well as study design as described in the manuscript, which make it hard to support the conclusions the authors describe in their discussion, at least in the current version of the manuscript.
My main concern is that the measurement conditions regarding the subject’s gaze direction seem very forced. A chin rest was used to constrict head movements, mentioned by the authors themselves as possible limitation. Additionally, the placement of the stimulus (at defined rotations for far vision and different blocks for near vision) seems to forces subjects to use a fixed gaze direction, sometimes probably through a blurry part of the lens (depending on design of the PPL). When forcing gaze through a blurry part of the lens, different visual performance between different designs seems quite obvious. The conclusions the authors make in the discussion might be misleading. It is well known that the coordination of head and eye movements changes for PPL wearers, so actual reading performance in a natural (not constricted) scenario might be different.
It is valid and interesting to compare different PPL designs regarding the visual performance in different defined gaze directions, but then results should be presented individually for each gaze direction and conclusions should be formulated more carefully. In this case it seems like authors combined measurements for different rotations. This should be described better in the manuscript, because this would definitely influence the results and seems very misleading to not describe it in detail in the method section.**

Responding to reviewer comment, A chin rest system was used to prevent head motion to assess how the visual performance in different gaze direction is affected by the different field of view of PPLs. The goal of the study was to evaluate how peripheral blur of the different PPLs affect eye movements and for that purpose 3 different PPLs with different fields of view has been used. Therefore, to assess the periphery of the lens it was necessary to ensure that all participants were looking in the same area to be studied, so a chin rest system was used. Regarding the analysis at different gaze directions, to simplify the results of the experiment the measures of each rotation were combined into one, although following the reviewers' recommendations we have modified the analysis and data for each rotation as can be seen in results section. To clarify this point, the following changes has been introduced throughout the manuscript:

Abstract section:

“The purpose of this study is to evaluate reading time and characteristics of fixations at different distances when looking through different areas of progressive power lenses (PPL) with different power distributions by means of eye-tracking technology.”

Introduction section:

“The main objective of this study is to evaluate and compare reading time and characteristics of fixations when looking through different areas of three PPLs with different power distributions at distance-reading and near-reading tasks.”

Methods section:

“*Distance-reading performance was recorded while reading a text shown on a screen monitor (Asus LCD Monitor VP228HE 21.5’’) located at 5.25m. The text subtended 4.2° horizontally and 2.3° vertically and was composed of 5 text lines having a font size of 0.4logMAR. To evaluate a greater area, subjects were rotated into 3 different orientations: on-axis and two off-axis reading positions (10° and 15°). Off-axis rotations were induced using a rotation platform with a chinrest to prevent head mobility (Fig 3). For each position, subjects were asked to read out loud the text displayed on the screen with the 3 PPLs. Therefore, nine reading texts for the different experimental conditions (3 PPLs and 3 rotations) were used in a randomized order.*

*Near-reading performance was assessed at 0.37m using 3 different reading texts (randomized for each PPL) displayed on a screen (Microsoft Surface PRO 4 12.3"). Each reading text was composed of three columns with five text lines each with a font size of 0.4logMAR and a viewing angle of 28° horizontally and 3.6° vertically. A table with a chinrest was used to prevent head movements and ensure subjects used the PPL central and lateral regions.”*

Results section:

“**Distance-reading vision**: Statistically significant differences were found between PPLs for distance-reading vision. When subjects were using a PPL optimized for distance vision, reading time; total duration of fixations and number of fixations decreased in comparison with PPL-Balance and PPL-Near. Statistically significant higher reading time and number of fixations were found for off-axis orientations in comparison with on-axis (Fig 5 and table 2).

Fig 5. Variations in reading time, fixation time and fixation count depending on the PPL (A), the orientation (B) and the interactions of PPL and orientation (C) for distance-reading task.

Table 2. Detailed statistics for Fig 5.

|  | **ANOVA test for lens design** | | | | **LSD Comparisons for lens design** | | |
| --- | --- | --- | --- | --- | --- | --- | --- |
|  | Df | Mean square | F-ratio | P-value | Balance/Near  (Diff limit) | Balance/Distance  (Diff+/-limit) | Distance/Near  (Diff+/-limit) |
| Reading time | 2 | 39.31 | 8.89 | <0.001* | -0.49*+/-*0.64 | 0.86*+/-*0.64* | -1.35*+/-*0.64* |
| Fixation time | 2 | 31.62 | 6.45 | 0.002* | -0.74*+/-*0.68* | 0.48*+/-*0.68 | -1.22*+/-*0.68* |
| Fixation count | 2 | 333.68 | 6.16 | 0.003* | -0.93*+/-*2.25 | 2.89*+/-*2.25* | -3.8*+/-*2.25* |
|  | **ANOVA test for orientation** | | | | **LSD comparisons for orientation** | | |
|  | Df | Mean square | F-ratio | P-value | On-axis/10º off-axis  (Diff+/-limit) | On-axis/15º off-axis  (Diff+/-limit) | 10 º/15 º off-axis  (Diff+/-limit) |
| Reading time | 2 | 21.47 | 4.86 | 0.01* | -0.69*+/-*0.64* | -0.98*+/-*0.64* | -0.29*+/-*0.64 |
| Fixation time | 2 | 0.39 | 0.08 | 0.924 | -0.13*+/-*0.68 | -0.09*+/-*0.68 | 0.04*+/-*0.68 |
| Fixation count | 2 | 1193.90 | 22.03 | <0.001* | -3.34*+/-*2.25* | -7.52*+/-*2.25* | -4.19*+/-*2.25* |

Multifactorial ANOVA test with pos-hoc comparisons using LSD. * Shows significance at the 0.05 level.

**Near-reading vision:** Statistically significant differences were found between PPLs for near-reading vision. When subjects were using a PPL optimized for near vision, reading time, total duration of fixations and the number of fixations decreased in comparison with PPL-Balance and PPL-Distance. Statistically significant lower reading time, lower fixation time and less fixation count were found for central column in comparison with peripheral columns (Fig 6 and table 3).

Fig 6. Variations in reading time, fixation time and fixation count depending on the PPL (A), the orientation (B) and the interactions of PPL and orientation (C) for near-reading task.

Table 3. Detailed statistics for Fig 6.

|  | **ANOVA test for lens design** | | | | **LSD Comparisons for lens design** | | |
| --- | --- | --- | --- | --- | --- | --- | --- |
|  | Df | Mean squeare | F-ratio | P-value | Balance/Near  (Diff+/-limit) | Balance/Distance  (Diff+/-limit) | Distance/Near  (Diff+/-limit) |
| Reading time | 2 | 135.30 | 12.24 | <0.001* | 1.64+/-0.81* | -0.86+/-0.81* | 2.50+/-0.81* |
| Fixation time | 2 | 54.27 | 7.79 | <0.001* | 0.97+/-0.58* | -0.63+/-0.58* | 1.60+/-0.58* |
| Fixation count | 2 | 1127.08 | 19.12 | <0.001* | 4.40+/-2.36* | -2.88+/-2.36* | 7.27+/-2.36* |
|  | **ANOVA test for reading columns** | | | | **LSD Comparisons for reading columns** | | |
|  | Df | Mean squeare | F-ratio | P-value | Center/Dominant  (Diff+/-limit) | Center/ Non-dominant  (Diff+/-limit) | Dominant/Non- dominant  (Diff+/-limit) |
| Reading time | 2 | 90.37 | 8.18 | <0.001* | -2.05+/-0.81* | -1.28+/-0.81* | 0.77+/-0.81 |
| Fixation time | 2 | 13.67 | 1.96 | 0.143 | -0.70+/-0.58* | -0.70+/-0.58* | 0.01+/-0.80 |
| Fixation count | 2 | 567.62 | 9.63 | <0.001* | -5.18+/-2.36* | -2.99+/-2.36* | 2.19+/-2.36 |

Multifactorial ANOVA test with pos-hoc comparisons using LSD. * Shows significance at the 0.05 level.

Discussion section:

“The results of this study show that when participants are induced to look through different areas of the lens, there are variations in reading time, total duration of fixations, and fixation count depending on the visual area of the PPL. A PPL design with a wider distance visual area provides lower reading time and less fixation count than PPL-Balance and PPL-Near, improving reading at distance vision. Similarly for near-vision reading, a PPL design with a wider near area provides lower reading time, lower total fixation time, and less fixation count than PPL-Balance and PPL-Distance, improving reading at near vision. Also, it has been observed the influence of peripheral distortions of the PPLs in reading performance. More specifically, reading time and fixation count was higher at off-axis positions in comparison with on-axis positions both at near and distance reading tasks.”

**Data results are only presented in a table as mean and standard deviation of each condition. I would recommend to at least present a Fig of the reading time results in the different conditions, ideally showing individual data points for each subject. The presented standard deviation is much higher than the difference of the mean values, so a Fig with individual data points might help making the differences between conditions understandable.**

According to reviewer suggestions, the result section has been improved including different graphics with mean values and its standard deviation.

**The authors describe their subjects to have quite a big range in refractive values. Yet the Fig they present for the power distribution of the different lens designs is only for one refractive value with (it seems like) far refraction 0 dpt. Is this the actual design of one of the subjects or is it a power distribution for a theoretical wearer with far refraction 0? The influence of the individual refraction in the far as well as near area needs to be discussed. A low addition value will lead to a smaller gradient in optical power throughout the lens and therefore to less unwanted optical aberrations. Showing a Fig with specific fisher values for the aberration is misleading, as it does not explain that those values can be quite different between different subjects. It is important to estimate if the difference between the subjects’ lenses is actually smaller than the differences between different designs. Otherwise it would be necessary to perform the analysis depending on the individual refraction to show how visual performance differences between different designs are influenced by the refractive value.**

**As shown in Fig 2, the PPLs were actually not placed in the spectacle frame but as a clip-on to the eye tracking glasses. In the methods it is described that “fitting parameters for the ET eyeglasses were measured”. Does this mean that the additional vertex distance with the clip-on was considered? How much is the vertex distance increased by such a setup compared to vertex distance with a typical frame? How does it influence the power distribution in the visual field? Is fig 1 calculated for this increased vertex distance?**

Responding reviewer’s comments, the Fig 2 corresponds to plano addition 2.00D where the lens has been assumed to have no tilt and with default parameters (such as refractive index of 1.5 or vertex distance of 14mm).

Regarding customization of lenses using free-form technology, when light rays from an object enter a lens at an angle, such as when a patient uses the lens' peripheral area to look away from the optical center or when the lenses are not perfectly perpendicular to the patient's viewing direction, oblique aberrations result. In these cases, the lens is essentially giving a prescription with the sphere, cylinder, and axis values set incorrectly, resulting in distorted vision and a reduction in the visual field. Recently, the process of making lenses has been improved with the irruption of free-form technology. Free-form technology enables the creation of arbitrary surfaces as opposed to conventional manufacturing procedures, which are limited to producing a sphere or torus on the back surface of the lens. Therefore, a much better compensation of the oblique aberrations is possible when spherical molded blanks are combined with a back surface that is manufactured point by point. Using advanced optical design software, it is possible to optimize the back lens surface to correct for oblique aberrations while considering the patient's position of wear (interpupilary distance, fitting height, back vertex distance, pantoscopic angle, wrapping angle, and near working distance), the lens' refractive index, prescription, base curve, and frame characteristics. The outcome is a user-compensated lens with a unique back surface customized for each patient's wearing position (1,2). So, this customization means that the design remains relatively stable and that there is no variation in the fields of view with the prescription addition or any customization parameter such us the back vertex distance of each subject as can be seen in the following tables where areas for distance and near region according to sheedy were analyzed(3).

|  | **Sheedy's area** | | | | | | **Difference compared to balance** | | | |
| --- | --- | --- | --- | --- | --- | --- | --- | --- | --- | --- |
|  | **PPL-Balance** | | **PPL-Distance** | | **PPL-Near** | | **Distance area (%)** | | **Near area (%)** | |
| ID | **Distance area(mm^2^)** | **Near area(mm^2^)** | **Distance area(mm^2^)** | **Near area(mm^2^)** | **Distance area(mm^2^)** | **Near area(mm^2^)** | **PPL-Distance** | **PPL-Near** | **PPL-Distance** | **PPL-Near** |
| 102 | 38,51 | 43,18 | 45,26 | 36,7 | 30,86 | 56,59 | 18% | -20% | -15% | 31% |
| 149 | 38,41 | 35,08 | 53,28 | 31,6 | 30,635 | 46,29 | 39% | -20% | -10% | 32% |
| 197 | 37,835 | 37,36 | 47,76 | 32,51 | 29,81 | 48,21 | 26% | -21% | -13% | 29% |
| 546 | 39,435 | 34,98 | 53,52 | 31,32 | 30,415 | 43,99 | 36% | -23% | -10% | 26% |
| 687 | 36,3 | 40,95 | 42,79 | 32,68 | 29,2 | 53,47 | 18% | -20% | -20% | 31% |
| 771 | 37,52 | 34,1 | 50,06 | 30,05 | 28,905 | 43,63 | 33% | -23% | -12% | 28% |
| 880 | 38,97 | 35,08 | 51,41 | 31,01 | 30,17 | 44,26 | 32% | -23% | -12% | 26% |
| 899 | 38,345 | 35,27 | 50,71 | 31,95 | 29,67 | 44,35 | 32% | -23% | -9% | 26% |
| 972 | 38,485 | 35,5 | 48,7 | 31,44 | 29,905 | 45,19 | 27% | -22% | -11% | 27% |
| 1692 | 38,04 | 35,37 | 49,24 | 31,06 | 29,37 | 44,99 | 29% | -23% | -12% | 27% |
| 1779 | 39,035 | 42,42 | 47,32 | 38,25 | 31,015 | 53,68 | 21% | -21% | -10% | 27% |
| 2200 | 39,23 | 39,49 | 47,035 | 35,9 | 31,035 | 50,53 | 20% | -21% | -9% | 28% |
| 2384 | 37,68 | 41,16 | 46,305 | 36,11 | 30,13 | 51,71 | 23% | -20% | -12% | 26% |
| 2404 | 38,625 | 35,07 | 51,11 | 31,06 | 29,96 | 44,2 | 32% | -22% | -11% | 26% |
| 2575 | 37,66 | 38,31 | 47,445 | 32,36 | 29,93 | 49,36 | 26% | -21% | -16% | 29% |
| 2855 | 36,455 | 32,29 | 47,305 | 28,51 | 28,34 | 41,26 | 30% | -22% | -12% | 28% |
| 2859 | 39,53 | 35,76 | 53,995 | 31,98 | 30,325 | 44,66 | 37% | -23% | -11% | 25% |
| 2865 | 38,755 | 35,7 | 50,545 | 32,16 | 30,03 | 45,09 | 30% | -23% | -10% | 26% |
| 2873 | 37,18 | 33,1 | 48,755 | 29,59 | 28,71 | 42,48 | 31% | -23% | -11% | 28% |
| 2916 | 39,09 | 41 | 49,98 | 37,25 | 30,32 | 52,19 | 28% | -22% | -9% | 27% |
| 2923 | 37,985 | 37,71 | 48,415 | 33,42 | 29,725 | 48,1 | 27% | -22% | -11% | 28% |
| 2943 | 38,21 | 36,58 | 49,35 | 33,39 | 29,845 | 46,18 | 29% | -22% | -9% | 26% |
| 2951 | 39,03 | 35,31 | 49,14 | 30,2 | 30,51 | 46,22 | 26% | -22% | -14% | 31% |
| 2954 | 38,685 | 38 | 48,085 | 33,1 | 30,345 | 48,07 | 24% | -22% | -13% | 26% |
| 2999 | 38,3 | 34,35 | 51,005 | 30,64 | 29,555 | 43,02 | 33% | -23% | -11% | 25% |
| 3037 | 37,375 | 33,86 | 48,255 | 30,18 | 29,02 | 43,04 | 29% | -22% | -11% | 27% |
| 3085 | 37,755 | 36,29 | 49,03 | 33,03 | 29,415 | 45,78 | 30% | -22% | -9% | 26% |
| 3088 | 40 | 37,61 | 53,175 | 33,57 | 31,18 | 47,03 | 33% | -22% | -11% | 25% |

*In the case of a regular frame the vertex distance is about 14mm, with the clip attached to the eye-tracker device all participants had a vertex distance between 18-20mm. It is true that there is an increase in the vertex distance, but in the calculation of the lenses this has been considered as well as the other customization parameters, thus there is no influence on visual fields.*

To clarify this in the manuscript, the sections on procedure and progressive lenses in methods has been modified as follows:

“**Procedure:** All subjects underwent a comprehensive optometric evaluation to check whether they meet the inclusion criteria. The visual exam consisted of VA testing using PVVAT test (Precision Vision, La Salle, III), subjective refraction at distance and near vision, stereoacuity evaluation using Titmus test, cover test, Worth test, and ocular motility analysis. Once optometrists confirmed the participant met the inclusion criteria, measurement of fitting parameters and position of wear (pupillary distance, segment height, pantoscopic tilt, back vertex distance, and frame wrap angle) for the ET eyeglasses with the clip-on attached were measured, and PPL lenses were ordered.”

**“Progressive Power Lenses:** Three free-form PPL designs individualized with different power distribution maps were developed ad-hoc for this research: 1) PPL-Balance, the control lens (Endless Steady Balance, IOT, Madrid, Spain), 2) PPL-Distance, a lens with a wider visual area for distance vision (Endless Steady Distance, IOT, Madrid, Spain) and 3) PPL-Near, a lens with wider visual area for near vision (Endless Steady Near, IOT, Madrid, Spain). Mean power, cylinder power maps distribution, Sheedy contours and visual areas according to Sheedy’s criteria (28) for a plano addition 2.00D prescription using standard parameters are shown in Fig 1.

Lenses were mounted in a specific clip-on (developed ad-hoc) that was attached to the front of the ET eyeglasses. This set up allowed the direct recording of the pupils without any interference from the progressive lenses. (Fig 2).

Fig 1: Mean power, cylinder power maps distribution, and visual areas according to Sheedy’s criteria for plano addition 2.00D prescription using standard parameters of the PPLs tested in the research A) PPL-Balance. B) PPL-Distance. C) PPL-Near

Fig 2: Picture of the metallic frame developed ad-hoc for this study attached to Tobii Glasses PRO 3.

*Lenses were calculated using fitting parameters and position of wear of the ET with the clip-on using advanced optical design software (Freeform designer, IOT, Madrid, Spain) to reduce oblique aberrations and maintain fields of view stable independent of each subject's prescription and/or addition power for all gaze directions. According to Sheddy's criteria, the PPL-Distance lens has a 29% wider field of view and the PPL-Near lens a 22% narrower field of view in the distance zone compared to the control lens (PPL-Balance). In the near zone, compared to the control lens, the PPL-Near has a 27% wider field of view and the PPL-Distance a 12% narrower field of view”*

**Line 132: “are shown in Fig 2.” Should be Fig. 1**

In response to reviewer comment, this has been corrected in the manuscript:

“Mean power, cylinder power maps distribution, Sheedy contours and visual areas according to Sheedy’s criteria (28) for a plano addition 2.00D prescription using standard parameters are shown in Fig 1.”

**How was reading time obtained from eye movements?**

In response to the reviewer's comment, for the calculation of eye movements a classification algorithm has been used that only considers saccades and fixations, therefore the reading time has been calculated as the sum of the fixation time and the saccade time (5,6)

**The supporting information file has the measurement data for each subject in far and near vision reading task and each PPL. But there are not the single measurements for the three different rotations in the far vision task shown. Where the measurements just combined?
In the near vision reading task, did subjects read each of the three blocks individually or was there only one combined measurement?**

Responding the reviewer comment, to simplify the results of the experiment the measures of each rotation at distance were combine into one. According to the reviewer’s recommendations we have included the analysis of three different rotations in the results section of the manuscript as previously shown. Regarding near-reading task, participants read a text with three different columns for each PPL. Then the analysis was split into three reading columns, previously called “text-blocks”. Dataset has been uploaded with all the different conditions.

**In table 2 all the units are seconds, in table 3 they are ms. I guess the authors mixed up different units for reading time and fixation time.**

In response to the reviewer, in table 3 the correct units are seconds, but according to the changes made in the results section these table have been deleted and in their place Fig 3 and Fig 4 have been included.

**Fig 1: Resolution could be improved. Also add titles for mean power and cyl power, units for the color bar. What is the FoV shown in the plots? Some labels for horizontal and vertical extent might help the reader to get an understanding for the size differences of far and near zones in visual angle (not only area in mm^2) between the different designs. I think it is important to understand the visual area covered by the stimulus in relation to the visual size of far and near zone.**

In response to the reviewer, Fig 1 has been improved following reviewer suggestions and it has been added how the area of the distance and near have been calculated as follows:

**The authors should differentiate better between optical distortions and blur/astigmatism. For example, in line 77 (“Unwanted astigmatism limits the area of the undistorted field of view of users, and its gradient is also responsible for swimming effects, unwanted distortion, and dizziness in dynamic visual conditions (23).”) and line 224 (“In other words, when undistorted visual areas are reduced, the user needs more time to read the text due to the increase of unwanted astigmatism in the lateral areas of the region used for reading.”) it seems like "undistorted area" is used equivalently to area with low astigmatism.**

Responding the reviewer comment, to be clearer we have reviewed the manuscript and we have tried to be consistent with the terminology, using the term field of view, when necessary, as the area of the lens with low distortion. Meaning low distortion as non-perceived distortion by users as has been defined in the document using the criteria from Sheedy.

References used in this letter:

1. Meister DJ, Fisher SW. Progress in the spectacle correction of presbyopia. Part 1: Design and development of progressive lenses. Vol. 91, Clinical and Experimental Optometry. 2008. p. 240–50.

2. Meister DJ, Fisher SW. Progress in the spectacle correction of presbyopia. Part 2: Modern progressive lens technologies. Vol. 91, Clinical and Experimental Optometry. 2008. p. 251–64.

3. Chamorro E, Alonso J, Cleva JM, Crespo D. Free-Form Lenses: Why My Patient is Not Wearing My Prescription. JOJ Ophthalmol. 2017;2(1):555577.

4. Sheedy JE. Correlation analysis of the optics of progressive addition lenses. Optometry and Vision Science. 2004 May;81(5):350–61.

5. Tobii Technology AB. Determining the Tobii I-VT fixation filter’s default values: method description and results discussion. 2012;

6. Olsen A. The Tobii I-VT fixation filter. Tobii Technology. 2012;21.
