# Supplementary material for: Correlation between reading time and characteristics of eye fixations and progressive lens design
Source: PLoS One. 2023 Mar 27;18(3):e0281861. doi: 10.1371/journal.pone.0281861 (PMC10042365; doi:10.1371/journal.pone.0281861)
Supplement: Attachment — Submitted filename: Response to Reviewers.docx [file pone.0281861.s003.docx]

**PABLO CONCEPCIÓN GRANDE M.Sc.**

Indizen Optical Technologies,SL.

Calle suero de quiñones 34-36, 3º

28005 MADRID, Spain

[pconcepcion@iot.es](mailto:pconcepcion@iot.es)

January 31^st^, 2023

Thanks to the reviewers and the editor for the revisions that have helped to improve this work significantly. Hereafter we provide replies to the points raised, and describe the changes made to the manuscript:

**Editor comment:**

**Thank you to the authors for addressing most of the reviewers' comments. Please address the remaining comment from the reviewer. Specifically, ensure that the term "distortions" is either explicitly defined or please use the term "refractive error" rather than "distortions" when refractive error is what is being referred to.**

In response to the editor, we have followed the suggestions of reviewer 2 and changed the term distortions to be more correct when referring to power errors by “refractive errors” in the following cases:

“Also, it has been observed the influence of peripheral refractive errors of the PPLs in reading performance.”

“In addition, our results show that when using the central area of the lens, with less refractive errors than the periphery, reading time, complete fixation time and fixation count decrease.”

**Journal Requirements:

Please review your reference list to ensure that it is complete and correct. If you have cited papers that have been retracted, please include the rationale for doing so in the manuscript text, or remove these references and replace them with relevant current references. Any changes to the reference list should be mentioned in the rebuttal letter that accompanies your revised manuscript. If you need to cite a retracted article, indicate the article’s retracted status in the References list and also include a citation and full reference for the retraction notice.**

In response to the journal's requirements, the list of bibliographic references has been reviewed and no changes have been made.

**Review Comments to the Author

Reviewer #2: The changes made by the authors in their analysis and manuscript vastly improved the study. The description of the procedure with different viewing directions is clearer now and the analysis depending also on this viewing direction makes more sense. Adding the figures 5 and 6 makes the results much clearer.

One last comment for changes:
Authors claim that they are now more consistent with terminology of “distortions”. I would still advice to not use the phrase “areas of low distortions” where it should mean “low refractive error/low astigmatism” throughout the manuscript. Optical distortions are not directly reflected by the distribution of spherical and cylindrical power.

“Meaning low distortion as non-perceived distortion by users as has been defined in the document using the criteria from Sheedy”

Also, the definition by Sheedy the authors are referring to does depend on the refractive error, not the optical distortions.**

In response to the reviewer, he is correct in that the Sheedy definition we referenced refers to refractive errors, not optical distortion. For this reason, the following changes have been made to the manuscript:

“Also, it has been observed the influence of peripheral refractive errors of the PPLs in reading performance.”

“In addition, our results show that when using the central area of the lens, with less refractive errors than the periphery, reading time, complete fixation time and fixation count decrease.”
