# Supplementary material for: Correlation between reading time and characteristics of eye fixations and progressive lens design
Source: PLoS One. 2023 Mar 27;18(3):e0281861. doi: 10.1371/journal.pone.0281861 (PMC10042365; doi:10.1371/journal.pone.0281861)
Supplement: Peer review history

# Peer review: Decision Letter 0

**DOI:** 10.1371/journal.pone.0281861.r001

**Authors:** Nick Fogt

14 Nov 2022

PONE-D-22-19980Correlation between reading time and characteristics of eye fixations and progressive lens design

PLOS ONE

Dear Dr. Concepcion-Grande,

Thank you for submitting your manuscript to PLOS ONE. After careful consideration, we feel that it has merit but does not fully meet PLOS ONE’s publication criteria as it currently stands. Therefore, we invite you to submit a revised version of the manuscript that addresses the points raised during the review process.

The reviewers both believe this paper has potential, but there are major concerns raised by reviewer #1 around the statistical analyses. Both reviewers raise the question of why the axis of rotation did not seem to be a focus of the paper. In addition, reviewer #2 raises a valid point as to the relevance of this study given that it is known that individuals adapt to their progressive addition lenses by altering their head movements. The authors should directly address what is to be gained from this study given that the results may (or may not) hold when individuals are able to move their heads as would normally be the case. Finally, both reviewers are concerned that overall there is a lack of description of the characteristics of the subjects. Please address every comment from both reviewers.

Please submit your revised manuscript by Dec 10 2022 11:59PM. If you will need more time than this to complete your revisions, please reply to this message or contact the journal office at plosone@plos.org. When you're ready to submit your revision, log on to https://www.editorialmanager.com/pone/ and select the 'Submissions Needing Revision' folder to locate your manuscript file.

Please include the following items when submitting your revised manuscript:

- A rebuttal letter that responds to each point raised by the academic editor and reviewer(s). You should upload this letter as a separate file labeled 'Response to Reviewers'.
- A marked-up copy of your manuscript that highlights changes made to the original version. You should upload this as a separate file labeled 'Revised Manuscript with Track Changes'.
- An unmarked version of your revised paper without tracked changes. You should upload this as a separate file labeled 'Manuscript'

If you would like to make changes to your financial disclosure, please include your updated statement in your cover letter. Guidelines for resubmitting your figure files are available below the reviewer comments at the end of this letter.

If applicable, we recommend that you deposit your laboratory protocols in protocols.io to enhance the reproducibility of your results. Protocols.io assigns your protocol its own identifier (DOI) so that it can be cited independently in the future. For instructions see: https://journals.plos.org/plosone/s/submission-guidelines#loc-laboratory-protocols. Additionally, PLOS ONE offers an option for publishing peer-reviewed Lab Protocol articles, which describe protocols hosted on protocols.io. Read more information on sharing protocols at https://plos.org/protocols?utm_medium=editorial-email&utm_source=authorletters&utm_campaign=protocols.

We look forward to receiving your revised manuscript.

Kind regards,

Nick Fogt

Academic Editor

PLOS ONE

Journal Requirements: 

When submitting your revision, we need you to address these additional requirements.

1. Please ensure that your manuscript meets PLOS ONE's style requirements, including those for file naming. The PLOS ONE style templates can be found at https://journals.plos.org/plosone/s/file?id=wjVg/PLOSOne_formatting_sample_main_body.pdf and 

https://journals.plos.org/plosone/s/file?id=ba62/PLOSOne_formatting_sample_title_authors_affiliations.pdf

2. We note that the grant information you provided in the ‘Funding Information’ and ‘Financial Disclosure’ sections do not match. 

When you resubmit, please ensure that you provide the correct grant numbers for the awards you received for your study in the ‘Funding Information’ section

​

Reviewers' comments:

Reviewer's Responses to Questions

**Comments to the Author**

1. Is the manuscript technically sound, and do the data support the conclusions?

The manuscript must describe a technically sound piece of scientific research with data that supports the conclusions. Experiments must have been conducted rigorously, with appropriate controls, replication, and sample sizes. The conclusions must be drawn appropriately based on the data presented. 

Reviewer #1: Partly

Reviewer #2: Partly

**********

2. Has the statistical analysis been performed appropriately and rigorously? 

Reviewer #1: No

Reviewer #2: I Don't Know

**********

3. Have the authors made all data underlying the findings in their manuscript fully available?

The PLOS Data policy requires authors to make all data underlying the findings described in their manuscript fully available without restriction, with rare exception (please refer to the Data Availability Statement in the manuscript PDF file). The data should be provided as part of the manuscript or its supporting information, or deposited to a public repository. For example, in addition to summary statistics, the data points behind means, medians and variance measures should be available. If there are restrictions on publicly sharing data—e.g. participant privacy or use of data from a third party—those must be specified.

Reviewer #1: No

Reviewer #2: Yes

**********

4. Is the manuscript presented in an intelligible fashion and written in standard English?

PLOS ONE does not copyedit accepted manuscripts, so the language in submitted articles must be clear, correct, and unambiguous. Any typographical or grammatical errors should be corrected at revision, so please note any specific errors here.

Reviewer #1: Yes

Reviewer #2: Yes

**********

5. Review Comments to the Author

Please use the space provided to explain your answers to the questions above. You may also include additional comments for the author, including concerns about dual publication, research ethics, or publication ethics. (Please upload your review as an attachment if it exceeds 20,000 characters)

Reviewer #1: Manuscript Number PONE-D-22-19980

1. IS THE MANUSCRIPT TECHNICALLY SOUND, AND DO THE DATA SUPPORT THE CONCLUSIONS? PARTLY

Design for Visual Tasks:

- The authors have measured the reading tasks at distance with a straight-ahead position, and two off-axis positions with the head turned 10deg and 15deg relative to the target [Lines 147-154]. Outcome variables of total reading time, total fixation time, and number of fixations were obtained. A derived variable of average fixation duration was also computed. Although the outcome variables were measured at the different orientations there were no data presented for the different orientations.

- In the results section, it appears that the outcome variables are reported as an average of the three positions with each lens – I was, at first, not clear why. The best description for why appears in the penultimate paragraph of the conclusion ([L257-263]) and I would suggest that this text is moved to the methods. Their analysis says “Nine text blocks for the different experimental conditions (3 PPLs and 3 rotations) were used in a randomized order.” [Line 151-152] and were analyzed using “…a randomized complete block statistical test.” [Line 178]; statistically I would interpret this as the angle of rotation being treated as a nuisance variable – I’m not sure if this was the author’s intention? Did the authors consider treating the angle of orientation as a dependent variable, from which the outcome variables might be described by an interaction between design and orientation relative to the target? This would give some validation for both the experimental design and the impact of peripheral lens areas on performance. Even if it is not included in the statistical analysis, descriptive results would be beneficial to their manuscript.

- For the near reading task the head position was not tilted relative to the text (“3 different text blocks (randomized for each PPL) displayed on a screen” [Line 157]). It became clearer here that the authors are using ‘blocks’ to describe experimental units, and the statistical analysis run appears to be a repeated measures ANOVA with PPL design as a factor. If this is the case, then it should be included in the description of the analysis.

Conclusions & Supporting Data

- The main conclusion that “…reading time and characteristics of fixations of PPL users are affected by the power distribution of the PPL…” [L:201-202] is quite general (and is supported, in general) but is a little bit superficial. More specifically, their study shows a difference between the distance and near PPL designs for some of the outcome variables, but neither differ from the balance PPL design in the majority of cases – which is not identified in the manuscript.

- The magnitude of astigmatism was not a variable in the model - other than being encompassed, very generally, by the design variable. It’s a little speculative to attribute the difference in performance entirely to unwanted astigmatism in the designs, rather than other reasons not reported such as the impact of the refractive error of the sample or the previous PPL design experience of the subjects. Not all subjects show exactly the same pattern of responses.

- I was a little bit surprised by the level of astigmatism in the peripheral zones of the lenses (for the plano +2.00 Add example; Figure 1), because the areas with ~1.75 or more astigmatism looks larger than I would have expected. I’m not sure if this arises because of the need to create a lens to go over the eye-tracker equipment, as the authors do say that the lenses are ‘…freeform…’ [L126] and are optimised for the position of wear parameters [L120-121]?

- There’s a portion of the discussion that refers to other research where the authors state “Changes during reading observed between different types of PPLs are similar to the differences found between good and poor readers.” [L 239-240]. This is correct in as much as the direction is the same – that is, the reading time is longer and the number of fixations are more numerous with the Distance PPL for the reading task than with the Near PPL. However, as a reader you come away with the sense that the magnitude of the difference is similar – i.e. you are a ‘poor’ reader with a Distance PPL compared with being a ‘good’ reader with a Near PPL. This would be an incorrect inference and is unclear for the reader. Bucci et al (for example) report a 2.6x increase in the number of fixations for dyslexic (‘poor’) versus non-dyslexic readers (‘good’) and ~2x fixation duration, whereas it is a 1.2x difference with a Distance PPL versus a Near PPL.

- [L237-238] “These results are consistent with our findings on reading skills being affected by the unwanted astigmatism…”. The results referred to would be consistent with unwanted astigmatism affecting ease of reading and supports the author’s conjecture that the unwanted astigmatism associated with the design is the culprit… but their experiment did not explicitly include astigmatism in the model, it included lens design; the astigmatism component is inferred.

- [L252-255] These sentences need additional clarity to indicate that the magnitude of impairment reported for ‘poor’ and ‘good’ readers is not the same as reported in their study.

2. HAS THE STATISTICAL ANALYSIS BEEN PERFORMED APPROPRIATELY AND RIGOROUSLY? NO

- Design of the PPL appears to be a main effect in their analysis. The authors report in the methods that “…PPL-Balance, the control lens …”. I was expecting, given its designation as a control lens, that the analysis might include contrasts between the PPL-Bal and the two lenses optimised for a specific distance (i.e. comparing the PPL-Distance design with the PPL-Balance and comparing the PPL-Near design with the PPL-Balance only).

- As it was not clear what analysis was run, I ran the data provided with a simple repeated measures ANOVA (with design as a factor) and got identical probability values as reported in Table 2 & Table 3. I was unable to confirm this was the same as the author’s analysis as neither F values or degrees of freedom were provided. Secondary to running the data, I noticed that many of the main effects have significant sphericity, so reporting of corrected probability values (Greenhouse-Geisser or Huynh-Feldt) is indicated. It does not change the significant/non-significant findings but is more precise.

- In the absence of contrasts, no post-hoc testing was reported on the main effect of PPL design, to indicate which designs yielded an average difference in performance. This may have been done (the authors report that [L188-189] “…was decreased in comparison with PPL-Balance and PPL-Near.”) but the probability values (or procedure) associated with the specific comparisons are not given. The omnibus probability value does not apply to the specific comparisons.

- Unless average fixation duration is a motivating variable of interest (it didn’t appear to be from the methods or the discussion), I don’t see the value of performing statistical testing on total fixation duration, fixation counts and average fixation duration (derived from fixation duration/fixation count). Shouldn’t it be total reading time and average fixation duration or total reading time, total fixation time, number of fixations? It is of value to see the data for average fixation duration, but I don’t think they have the experimental power (or the experimental intention) to run 3 separate ANOVAs on the same data.

3. HAVE THE AUTHORS MADE ALL DATA UNDERLYING THE FINDINGS IN THEIR MANUSCRIPT FULLY AVAILABLE? NO

- The authors give their inclusion/exclusion criteria, but do not give the reader a comprehensive description of (a) the refractive errors for their sample, or (b) the general design characteristics (i.e balanced, distance or near PPL) worn by their subjects for at least 6 months prior to the study.

- (a) Although the authors provide a representative power map for Plano with +2.00D Add for each lens design, I didn’t have a sense of how representative this was for the study sample. The authors do report the number of subjects with myopia/ hyperopia/ emmetropia and the range of Add powers [L 182-185], but do not indicate these characteristics by subject in their dataset. In general, PPLs will show a reduction in area and increased astigmatic blur with increasing Add power and the areas of clear vision may also be affected by the distance refractive error. Given that the discussion relies heavily on the impact of astigmatism from the less optimal areas of the lens, this seems to be relevant data to support their conclusions.

- (b) This might give some more practical insight into any observed changes in duration/number of fixations. For example, were the subjects that showed much longer times for the distance reading tasks with the distance or near PPL, those that were wearing a balance PPL prior to the study? Or were the longer times associated with subjects that had higher amounts of refractive cylinder? Or higher addition powers? Etc

4. IS THE MANUSCRIPT PRESENTED IN AN INTELLIGIBLE FASHION AND WRITTEN IN STANDARD ENGLISH? YES

Specific typographical/grammatical suggestions:

[L64-67] [L82-86] Please move reference citation numbers to after the authors name, rather than the end of the sentences.

[L91-93] “The main objective of this study is to evaluate and compare reading time and characteristics of fixations when using three PPLs with different power distributions at distance-reading and near-reading tasks.” Suggest change to “… when performing a distance-reading and a near-reading task.”

[L96-97] “…carried out to evaluate characteristics of fixations when reading…”. Suggest change to “…carried out to evaluate reading time and characteristics of fixations when reading…”.

[L99] Should the ethics approval number be included?

[L100] Is it two pairs of eyeglasses? Or two lenses?

[L102] “…and with previous experience of at least 6 months wearing PPLs.” Suggest change to “…and with at least 6 months prior experience of PPL wear.”

[L106] “…binocular 4) Anisometropia…” is missing punctuation

[L118] I believe ‘worth’ should be capitalized in “… worth test…”

[L125] “…between each measurement were done to avoid participants’ fatigue.”. Suggest ‘minimize’ be used instead of ‘avoid’.

[L132] The power distribution maps are in Figure 1; Figure 2 is referenced in the text.

[L178] “…randomized complete block statistical test.” Suggest substituting repeated measures ANOVA for ‘statistical test’.

[L180] See the statistical analysis comments for the inclusion of the statistical analysis of average duration of fixations.

[L185] “The addition of the participants…” Suggest changing to “The addition power for the participants…”

[L204] “…improving reading skills…” Consider replacing skills (which implies more than reading speed/fixations) with a more representative word.

[L211-212] “…subject’s midline at 0.6m, while their head was fixed with a chinrest.” Remove ‘while their head was fixed with a chinrest’ as the study analyzed eye and head movements without a chinrest.

[L240] “…good and poor readers.. Bucci et al….” Remove extra period.

[L264] “…more natural condition it would…” Change condition to conditions.

[L265-267] “On the other hand, it would be interesting the evaluation of reading performance and ocular movements without the restrictions of using chinrest to avoid head movements.” Suggest changing to “Similarly, it would be interesting to evaluate reading performance and ocular movements without the restriction of a chinrest.”

[L269] “…with different power distributions at distance-reading…”. Suggest changing ‘at’ to ‘for’.

The references, in general, need some work. They are inconsistent with each other – for example: some have full journal titles, some abbreviated; some have year only, others have year and month, and others have year, month and day. Reference #s 4, 5, 7, 8, 9, 12, 16, 17, 21, 22, and 23 appear to be incomplete.

Reviewer #2: The manuscript describes a study using eye tracking to compare visual performance (tested in a reading task) with different progressive lenses. Focus of the study is on comparing lens designs optimized either for more far or more near vision tasks. The topic is very interesting and might have interesting results for ophthalmologists, optics designers and vision scientists. Nevertheless, I see some problems with the data analysis as well as study design as described in the manuscript, which make it hard to support the conclusions the authors describe in their discussion, at least in the current version of the manuscript.

My main concern is that the measurement conditions regarding the subject’s gaze direction seem very forced. A chin rest was used to constrict head movements, mentioned by the authors themselves as possible limitation. Additionally, the placement of the stimulus (at defined rotations for far vision and different blocks for near vision) seems to forces subjects to use a fixed gaze direction, sometimes probably through a blurry part of the lens (depending on design of the PPL). When forcing gaze through a blurry part of the lens, different visual performance between different designs seems quite obvious. The conclusions the authors make in the discussion might be misleading. It is well known that the coordination of head and eye movements changes for PPL wearers, so actual reading performance in a natural (not constricted) scenario might be different.

It is valid and interesting to compare different PPL designs regarding the visual performance in different defined gaze directions, but then results should be presented individually for each gaze direction and conclusions should be formulated more carefully. In this case it seems like authors combined measurements for different rotations. This should be described better in the manuscript, because this would definitely influence the results and seems very misleading to not describe it in detail in the method section.

Data results are only presented in a table as mean and standard deviation of each condition. I would recommend to at least present a figure of the reading time results in the different conditions, ideally showing individual data points for each subject. The presented standard deviation is much higher than the difference of the mean values, so a figure with individual data points might help making the differences between conditions understandable.

The authors describe their subjects to have quite a big range in refractive values. Yet the figure they present for the power distribution of the different lens designs is only for one refractive value with (it seems like) far refraction 0 dpt. Is this the actual design of one of the subjects or is it a power distribution for a theoretical wearer with far refraction 0? The influence of the individual refraction in the far as well as near area needs to be discussed. A low addition value will lead to a smaller gradient in optical power throughout the lens and therefore to less unwanted optical aberrations. Showing a figure with specific fisher values for the aberration is misleading, as it does not explain that those values can be quite different between different subjects. It is important to estimate if the difference between the subjects’ lenses is actually smaller than the differences between different designs. Otherwise it would be necessary to perform the analysis depending on the individual refraction to show how visual performance differences between different designs are influenced by the refractive value.

As shown in figure 2, the PPLs were actually not placed in the spectacle frame but as a clip-on to the eye tracking glasses. In the methods it is described that “fitting parameters for the ET eyeglasses were measured”. Does this mean that the additional vertex distance with the clip-on was considered? How much is the vertex distance increased by such a setup compared to vertex distance with a typical frame? How does it influence the power distribution in the visual field? Is fig 1 calculated for this increased vertex distance?

Line 132: “are shown in Fig 2.” Should be Fig. 1

How was reading time obtained from eye movements?

The supporting information file has the measurement data for each subject in far and near vision reading task and each PPL. But there are not the single measurements for the three different rotations in the far vision task shown. Where the measurements just combined?

In the near vision reading task, did subjects read each of the three blocks individually or was there only one combined measurement?

In table 2 all the units are seconds, in table 3 they are ms. I guess the authors mixed up different units for reading time and fixation time.

Fig 1: Resolution could be improved. Also add titles for mean power and cyl power, units for the color bar. What is the FoV shown in the plots? Some labels for horizontal and vertical extent might help the reader to get an understanding for the size differences of far and near zones in visual angle (not only area in mm^2) between the different designs. I think it is important to understand the visual area covered by the stimulus in relation to the visual size of far and near zone.

The authors should differentiate better between optical distortions and blur/astigmatism. For example, in line 77 (“Unwanted astigmatism limits the area of the undistorted field of view of users, and its gradient is also responsible for swimming effects, unwanted distortion, and dizziness in dynamic visual conditions (23).”) and line 224 (“In other words, when undistorted visual areas are reduced, the user needs more time to read the text due to the increase of unwanted astigmatism in the lateral areas of the region used for reading.”) it seems like "undistorted area" is used equivalently to area with low astigmatism.

**********

6. PLOS authors have the option to publish the peer review history of their article (what does this mean?). If published, this will include your full peer review and any attached files.

If you choose “no”, your identity will remain anonymous but your review may still be made public.

**Do you want your identity to be public for this peer review?** For information about this choice, including consent withdrawal, please see our Privacy Policy.

Reviewer #1: No

Reviewer #2: **Yes: **Yannick Sauer

**********

[NOTE: If reviewer comments were submitted as an attachment file, they will be attached to this email and accessible via the submission site. Please log into your account, locate the manuscript record, and check for the action link "View Attachments". If this link does not appear, there are no attachment files.]

While revising your submission, please upload your figure files to the Preflight Analysis and Conversion Engine (PACE) digital diagnostic tool, https://pacev2.apexcovantage.com/. PACE helps ensure that figures meet PLOS requirements. To use PACE, you must first register as a user. Registration is free. Then, login and navigate to the UPLOAD tab, where you will find detailed instructions on how to use the tool. If you encounter any issues or have any questions when using PACE, please email PLOS at figures@plos.org. Please note that Supporting Information files do not need this step.

# Author comment: Author response to Decision Letter 0

**DOI:** 10.1371/journal.pone.0281861.r002

8 Dec 2022

I have tried to follow all the comments and suggestions provided by the reviewers and the editor. In the file "Response to reviewers" you can find all the information related to this. 

If you have any additional questions about any of the documents submitted in this review, please do not hesitate to contact me.

Thank you very much for reviewing the manuscript and the changes made in this second submission.

Pablo Concepcion-Grande

Attachment Submitted filename: Response to Reviewers.docx Click here for additional data file.

# Peer review: Decision Letter 1

**DOI:** 10.1371/journal.pone.0281861.r003

**Authors:** Nick Fogt

30 Jan 2023

PONE-D-22-19980R1Correlation between reading time and characteristics of eye fixations and progressive lens designPLOS ONE

Dear Dr. Concepcion-Grande,

Thank you for submitting your manuscript to PLOS ONE. After careful consideration, we feel that it has merit but does not fully meet PLOS ONE’s publication criteria as it currently stands. Therefore, we invite you to submit a revised version of the manuscript that addresses the points raised during the review process.

==============================Thank you to the authors for addressing most of the reviewers' comments. Please address the remaining comment from the reviewer. Specifically, ensure that the term "distortions" is either explicitly defined or please use the term "refractive error" rather than "distortions" when refractive error is what is being referred to.

==============================

Please submit your revised manuscript by Mar 16 2023 11:59PM. If you will need more time than this to complete your revisions, please reply to this message or contact the journal office at plosone@plos.org. When you're ready to submit your revision, log on to https://www.editorialmanager.com/pone/ and select the 'Submissions Needing Revision' folder to locate your manuscript file.

Please include the following items when submitting your revised manuscript:

- A rebuttal letter that responds to each point raised by the academic editor and reviewer(s). You should upload this letter as a separate file labeled 'Response to Reviewers'.
- A marked-up copy of your manuscript that highlights changes made to the original version. You should upload this as a separate file labeled 'Revised Manuscript with Track Changes'.
- An unmarked version of your revised paper without tracked changes. You should upload this as a separate file labeled 'Manuscript'.

If you would like to make changes to your financial disclosure, please include your updated statement in your cover letter. Guidelines for resubmitting your figure files are available below the reviewer comments at the end of this letter.

If applicable, we recommend that you deposit your laboratory protocols in protocols.io to enhance the reproducibility of your results. Protocols.io assigns your protocol its own identifier (DOI) so that it can be cited independently in the future. For instructions see: https://journals.plos.org/plosone/s/submission-guidelines#loc-laboratory-protocols. Additionally, PLOS ONE offers an option for publishing peer-reviewed Lab Protocol articles, which describe protocols hosted on protocols.io. Read more information on sharing protocols at https://plos.org/protocols?utm_medium=editorial-email&utm_source=authorletters&utm_campaign=protocols.

We look forward to receiving your revised manuscript.

Kind regards,

Nick Fogt

Academic Editor

PLOS ONE

Journal Requirements:

Please review your reference list to ensure that it is complete and correct. If you have cited papers that have been retracted, please include the rationale for doing so in the manuscript text, or remove these references and replace them with relevant current references. Any changes to the reference list should be mentioned in the rebuttal letter that accompanies your revised manuscript. If you need to cite a retracted article, indicate the article’s retracted status in the References list and also include a citation and full reference for the retraction notice.

[Note: HTML markup is below. Please do not edit.]

Reviewers' comments:

Reviewer's Responses to Questions

**Comments to the Author**

1. If the authors have adequately addressed your comments raised in a previous round of review and you feel that this manuscript is now acceptable for publication, you may indicate that here to bypass the “Comments to the Author” section, enter your conflict of interest statement in the “Confidential to Editor” section, and submit your "Accept" recommendation.

Reviewer #2: (No Response)

**********

2. Is the manuscript technically sound, and do the data support the conclusions?

The manuscript must describe a technically sound piece of scientific research with data that supports the conclusions. Experiments must have been conducted rigorously, with appropriate controls, replication, and sample sizes. The conclusions must be drawn appropriately based on the data presented. 

Reviewer #2: Yes

**********

3. Has the statistical analysis been performed appropriately and rigorously? 

Reviewer #2: Yes

**********

4. Have the authors made all data underlying the findings in their manuscript fully available?

The PLOS Data policy requires authors to make all data underlying the findings described in their manuscript fully available without restriction, with rare exception (please refer to the Data Availability Statement in the manuscript PDF file). The data should be provided as part of the manuscript or its supporting information, or deposited to a public repository. For example, in addition to summary statistics, the data points behind means, medians and variance measures should be available. If there are restrictions on publicly sharing data—e.g. participant privacy or use of data from a third party—those must be specified.

Reviewer #2: Yes

**********

5. Is the manuscript presented in an intelligible fashion and written in standard English?

PLOS ONE does not copyedit accepted manuscripts, so the language in submitted articles must be clear, correct, and unambiguous. Any typographical or grammatical errors should be corrected at revision, so please note any specific errors here.

Reviewer #2: Yes

**********

6. Review Comments to the Author

Please use the space provided to explain your answers to the questions above. You may also include additional comments for the author, including concerns about dual publication, research ethics, or publication ethics. (Please upload your review as an attachment if it exceeds 20,000 characters)

Reviewer #2: The changes made by the authors in their analysis and manuscript vastly improved the study. The description of the procedure with different viewing directions is clearer now and the analysis depending also on this viewing direction makes more sense. Adding the figures 5 and 6 makes the results much clearer.

One last comment for changes:

Authors claim that they are now more consistent with terminology of “distortions”. I would still advice to not use the phrase “areas of low distortions” where it should mean “low refractive error/low astigmatism” throughout the manuscript. Optical distortions are not directly reflected by the distribution of spherical and cylindrical power.

“Meaning low distortion as non-perceived distortion by users as has been defined in the document using the criteria from Sheedy”

Also, the definition by Sheedy the authors are referring to does depend on the refractive error, not the optical distortions.

**********

7. PLOS authors have the option to publish the peer review history of their article (what does this mean?). If published, this will include your full peer review and any attached files.

If you choose “no”, your identity will remain anonymous but your review may still be made public.

**Do you want your identity to be public for this peer review?** For information about this choice, including consent withdrawal, please see our Privacy Policy.

Reviewer #2: No

**********

[NOTE: If reviewer comments were submitted as an attachment file, they will be attached to this email and accessible via the submission site. Please log into your account, locate the manuscript record, and check for the action link "View Attachments". If this link does not appear, there are no attachment files.]

While revising your submission, please upload your figure files to the Preflight Analysis and Conversion Engine (PACE) digital diagnostic tool, https://pacev2.apexcovantage.com/. PACE helps ensure that figures meet PLOS requirements. To use PACE, you must first register as a user. Registration is free. Then, login and navigate to the UPLOAD tab, where you will find detailed instructions on how to use the tool. If you encounter any issues or have any questions when using PACE, please email PLOS at figures@plos.org. Please note that Supporting Information files do not need this step.

# Author comment: Author response to Decision Letter 1

**DOI:** 10.1371/journal.pone.0281861.r004

31 Jan 2023

I have tried to follow all the comments and suggestions provided by the reviewers and the editor. In the file "Response to reviewers" you can find all the information related to this.

If you have any additional questions about any of the documents submitted in this review, please do not hesitate to contact me.

Thank you very much for reviewing the manuscript and the changes made in this new submission.

Pablo Concepcion-Grande

Attachment Submitted filename: Response to Reviewers.docx Click here for additional data file.

# Editor report: Decision Letter 2

**DOI:** 10.1371/journal.pone.0281861.r005

**Authors:** Nick Fogt

3 Feb 2023

Correlation between reading time and characteristics of eye fixations and progressive lens design

PONE-D-22-19980R2

Dear Dr. Concepcion-Grande,

We’re pleased to inform you that your manuscript has been judged scientifically suitable for publication and will be formally accepted for publication once it meets all outstanding technical requirements.

Within one week, you’ll receive an e-mail detailing the required amendments. When these have been addressed, you’ll receive a formal acceptance letter and your manuscript will be scheduled for publication.

An invoice for payment will follow shortly after the formal acceptance. To ensure an efficient process, please log into Editorial Manager at http://www.editorialmanager.com/pone/, click the 'Update My Information' link at the top of the page, and double check that your user information is up-to-date. If you have any billing related questions, please contact our Author Billing department directly at authorbilling@plos.org.

If your institution or institutions have a press office, please notify them about your upcoming paper to help maximize its impact. If they’ll be preparing press materials, please inform our press team as soon as possible -- no later than 48 hours after receiving the formal acceptance. Your manuscript will remain under strict press embargo until 2 pm Eastern Time on the date of publication. For more information, please contact onepress@plos.org.

Kind regards,

Nick Fogt

Academic Editor

PLOS ONE

Additional Editor Comments (optional):

Thank you for your detailed responses to the reviewer comments. The paper is ready for acceptance.

Reviewers' comments:

# Editor report: Acceptance letter

**DOI:** 10.1371/journal.pone.0281861.r006

**Authors:** Nick Fogt

17 Mar 2023

PONE-D-22-19980R2 

Correlation between reading time and characteristics of eye fixations and progressive lens design 

Dear Dr. Concepcion-Grande:

I'm pleased to inform you that your manuscript has been deemed suitable for publication in PLOS ONE. Congratulations! Your manuscript is now with our production department. 

If your institution or institutions have a press office, please let them know about your upcoming paper now to help maximize its impact. If they'll be preparing press materials, please inform our press team within the next 48 hours. Your manuscript will remain under strict press embargo until 2 pm Eastern Time on the date of publication. For more information please contact onepress@plos.org.

If we can help with anything else, please email us at plosone@plos.org. 

Thank you for submitting your work to PLOS ONE and supporting open access. 

Kind regards, 

PLOS ONE Editorial Office Staff

on behalf of

Dr. Nick Fogt 

Academic Editor

PLOS ONE
